# Differential phenotyping of *Brucella *species using a newly developed semi-automated metabolic system

**DOI:** 10.1186/1471-2180-10-269

**Published:** 2010-10-23

**Authors:** Sascha Al Dahouk, Holger C Scholz, Herbert Tomaso, Peter Bahn, Cornelia Göllner, Wolfram Karges, Bernd Appel, Andreas Hensel, Heinrich Neubauer, Karsten Nöckler

**Affiliations:** 1 Federal Institute for Risk Assessment, Diedersdorfer Weg 1, D-12277 Berlin, Germany; 2 RWTH Aachen University, Department of Internal Medicine III, Pauwelsstraße 30, D-52074 Aachen, Germany; 3 Bundeswehr Institute of Microbiology, Department of Bacteriology, Neuherbergstraße 11, D-80937 Munich, Germany; 4 Friedrich Loeffler Institute, Institute of Bacterial Infections and Zoonoses, Naumburgerstraße 96a, D-07743 Jena, Germany

## Abstract

**Background:**

A commercial biotyping system (Taxa Profile™, Merlin Diagnostika) testing the metabolization of various substrates by bacteria was used to determine if a set of phenotypic features will allow the identification of members of the genus *Brucella *and their differentiation into species and biovars.

**Results:**

A total of 191 different amines, amides, amino acids, other organic acids and heterocyclic and aromatic substrates (Taxa Profile™ A), 191 different mono-, di-, tri- and polysaccharides and sugar derivates (Taxa Profile™ C) and 95 amino peptidase- and protease-reactions, 76 glycosidase-, phosphatase- and other esterase-reactions, and 17 classic reactions (Taxa Profile™ E) were tested with the 23 reference strains representing the currently known species and biovars of *Brucella *and a collection of 60 field isolates. Based on specific and stable reactions a 96-well "*Brucella *identification and typing" plate (Micronaut™) was designed and re-tested in 113 *Brucella *isolates and a couple of closely related bacteria.

*Brucella *species and biovars revealed characteristic metabolic profiles and each strain showed an individual pattern. Due to their typical metabolic profiles a differentiation of *Brucella *isolates to the species level could be achieved. The separation of *B. canis *from *B. suis *bv 3, however, failed. At the biovar level, *B. abortus *bv 4, 5, 7 and *B. suis *bv 1-5 could be discriminated with a specificity of 100%. *B. melitensis *isolates clustered in a very homogenous group and could not be resolved according to their assigned biovars.

**Conclusions:**

The comprehensive testing of metabolic activity allows cluster analysis within the genus *Brucella*. The biotyping system developed for the identification of *Brucella *and differentiation of its species and biovars may replace or at least complement time-consuming tube testing especially in case of atypical strains. An easy to handle identification software facilitates the applicability of the Micronaut™ system for microbiology laboratories.

## Background

*Brucella *spp. are the causative agents of brucellosis, one of the major bacterial zoonotic diseases that is responsible for reproductive failure in animals leading to tremendous economic losses and for a potentially debilitating infection in man. Furthermore, *Brucella *is listed as category B bioterrorism agent.

Species and biovar classification of brucellae is historically based on natural host preference and phenotypic traits, i.e. CO_2 _requirement, H_2_S production, urease activity, dye-sensitivity, lysis by *Brucella*-specific bacteriophages, agglutination with monospecific antisera, and oxidative metabolic patterns [1-3]. In concordance with this biotyping scheme the genus *Brucella *(*B.*) currently comprises the six classical species *B. melitensis *bv 1-3 (predominantly isolated from sheep and goats), *B. abortus *bv 1-7 and 9 (from cattle and other Bovidae), *B. suis *bv 1-3 (from pigs), bv 4 (from reindeer) and bv 5 (from small ruminants), *B. canis *(from dogs), *B. ovis *(from sheep), and *B. neotomae *(from desert wood rats) [4]. Further, two novel species of marine origin, *B. pinnipedialis *(from seals) and *B. ceti *(from dolphins and whales) [5], and *B. microti *at first isolated from the common vole *Microtus arvalis *[6], then from red foxes (*Vulpes vulpes*) [7] and also directly from soil [8] have been added to the genus. Most recently *B. inopinata *sp. nov. isolated from a breast implant wound of a female patient has been described as a new species with so far unknown animal reservoir [9].

A biotyping assay useful for *Brucella *identification and species differentiation must consequently be able to identify the rising number of upcoming new species as well as single atypical strains which do not fit within the pre-existing scheme [10,11]. In addition, clinically relevant and closely related bacteria of other genera should be discriminated. Using commercially available rapid bacterial identification systems such as the API 20 NE^® ^(BioMerieux, Nürtingen, Germany) which include a restricted number of biochemical tests *Brucella *spp. may be misidentified e.g. as *Psychrobacter phenylpyruvicus *(formerly *Moraxella phenylpyruvica*) [12] or *Ochrobactrum anthropi *[13].

The aim of our study was to develop a miniaturised semi-automated system for the reliable identification of members of the genus *Brucella *and the differentiation of its species based on comprehensive metabolic activity testing.

## Results

**Figure 1:**
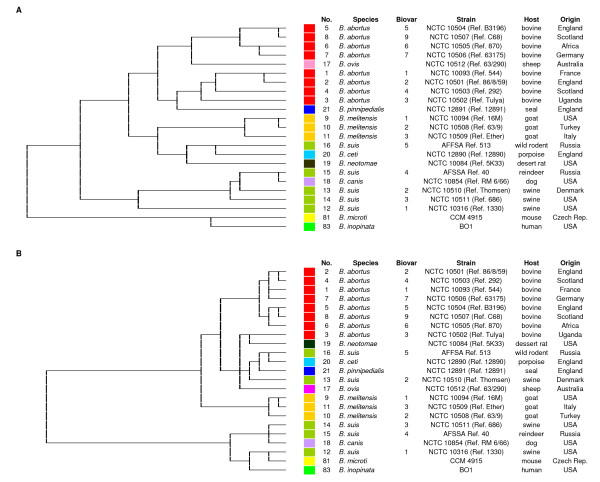
**Cluster analysis of *Brucella *reference strains based on biochemical reactions**. Cluster analysis of the 23 *Brucella *reference strains based on 570 (A) and 93 (B) biochemical reactions tested with the Taxa Profile™ system (plate A, C, and E) and the newly developed *Brucella *specific Micronaut™ microtiter plate, respectively. Hierarchical cluster analysis was performed by the Ward's linkage algorithm using the binary coded data based on the empirically set cut-off.

The Taxa Profile™ system testing the utilization of amino acids (A plates) and carbohydrates (C plates) as well as other enzymatic reactions (E plates) [Additional files 1, 2 and 3] revealed a very high biodiversity among the closely related species and biovars of the genus *Brucella *(Figure 1A, [Additional files 4, 5 and 6] ). The stability of metabolic profiles significantly varied between the different species and biovars, yet most of the stable markers were found in the Taxa Profile™ E plate. Differences between cultures of the same strain were most frequently observed in the species *B. abortus *and *B. microti*, and in biovar 1 of *B. suis*. A total of 196 out of 570 biochemical reactions proved to be both stable and discriminatory, and showed differences in the metabolism of the 23 *Brucella *reference strains or helped to distinguish *Brucella *spp. from closely related bacteria such as *Ochrobactrum *spp. In general, the broadest metabolic activity could be observed for strains of the species *B. suis*, *B. microti*, and *B. inopinata*. In contrast, the metabolic activity of *B. ovis*, *B. neotomae *and *B. pinnipedialis *was low.

**Figure 2:**
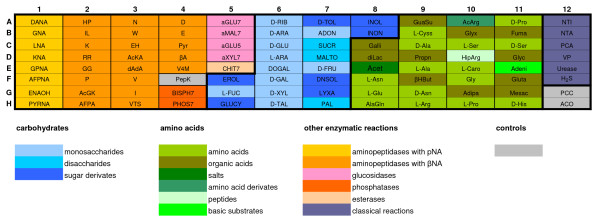
**The *Brucella *specific Micronaut™ microtiter plate**. Design of the newly developed *Brucella *specific Micronaut™ microtiter plate including 93 selected substances.

The comprehensive biotyping of the reference strains resulted in clusters agreeing in principle with the present conception of the genus *Brucella *(Figure 1A). A subset of 93 substances which preserved the clustering of the reference strains and achieved a satisfying discrimination was consecutively selected (Figures 2 and 1B). The newly configured 96-well plate assay tested for 29 aminopeptidases, 2 phosphatases, 4 glucosidases, 1 esterase, and the metabolism of 11 monosaccharides, 3 disaccharides, 7 sugar derivates, 15 amino acids, 11 organic acids, 1 salt, 1 amino acid derivate, 1 peptide, and 1 base. In addition, 6 classical reactions, i.e. nitrite, nitrate, pyrazinamidase, Voges-Proskauer medium, urease and H_2_S production, and three controls, i.e. peptidase control, pyrazinamidase control and assimilation control were included.

Glu(pNA)-OH (ENAOH), Pyr-pNA (PYRNA) (constantly negative reaction), and H-hydroxyprolin-βNA (HP) (constantly strong positive reaction) turned out to be key substances useful for the identification of the genus *Brucella *and its differentiation from other bacteria [Additional file 7].

A stable negative reaction for D-threitol (D-TOL) and mostly positive reactions for L-alanine (L-Ala), D-alanine (D-Ala), propionic acid (Propn), L-proline (L-Pro), D-proline (D-Pro), and D-serine (D-Ser) could be observed in *B. melitensis*. *B. microti *which also makes use of alanine and proline could be separated from *B. melitensis *by a constantly negative reactivity for Propn and D-Ser. A positive myo-inositol (INOL) reaction seemed to be characteristic for most *B. melitensis *strains and *B. inopinata*. Bis-p-nitrophenyl phosphate pH 7.5 (BISPH7), p-nitrophenyl phosphate di(2-amino-2-ethyl-1,3-propanediol) pH 7.5 (PHOS7), and p-nitrophenyl-a-d-glucopyranoside pH 7.5 (aGLU7) were found positive frequently in *B. suis *and regularly in *B. microti *strains, variable in *B. melitensis *and mostly negative in *B. abortus*. Glutarate (Gluta) and mesaconic acid (Mesac) which were almost exclusively metabolized by *B. microti *might be helpful for further differentiation. P-nitrophenyl-a-d-glucopyranoside pH 5.5 (aGLU5) and p-nitrophenyl-n-acetyl-β-d-glucosaminide pH 7.5 (CHIT7) showed weak positive reactions in *B. suis *and *B. canis *and strong positive reactions in *B. microti *and *B. inopinata*. *B. microti *and *B. inopinata *exhibited outstanding metabolic capabilities in comparison to all other brucellae, sharing a series of reactions with *O. anthropi *and *O. intermedium*. Most remarkably, both species were strongly positive in the Voges-Proskauer reaction. The slow growing strains of the *B. ovis *group did not metabolize any carbohydrates except for D-glucose-L-cysteine (GLUCY), L(+)-arabinose (L-ARA), D-TOL, and adonite (ADON) and only a few amino acids. In addition, *B. ovis *strains were usually not able to deoxidize nitrite (NTI, nitrite reduction) and nitrate (NTA, nitrate reduction). Ac-Gly-Lys-βNA (AcGK) tested strongly positive in *B. ovis *and *B. canis *whereas Trp-βNA (W) regularly tested negative in these species as compared to all other *Brucella *spp. In comparison with other species *B. pinnipedialis *was weak in metabolizing D(-)-ribose (D-RIB), D(-)-arabinose (D-ARA), D(+)-glucose (D-GLU), L-ARA, D(+)-galactose (D-GAL), D(+)-xylose (D-XYL), and a-D-talose (D-TAL). *B. ceti *and *B. pinnipedialis *showed significantly different carbohydrate utilization patterns. *B. neotomae *was the only species tested negative for d-Ala-pNA (DANA), Gly-pNA (GNA), Leu-pNA (LNA), Lys-pNA (KNA), Lys-βNA (K), and Gly-Gly-βNA (GG). Like *B. neotomae *the two yet unidentified strains isolated from foxes were negative for DANA and GNA. Despite of genetic consistency with the genus *Brucella *(*data not shown*) these two strains completely differed in their metabolic profile from the species described to date.

**Figure 3:**
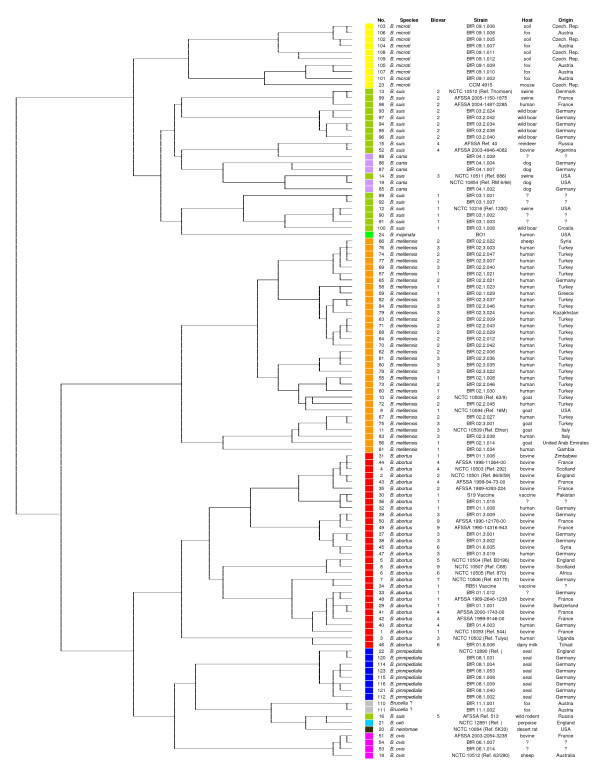
**Cluster analysis of *Brucella *field isolates based on biochemical reactions**. Cluster analysis of 113 *Brucella *strains including the reference strains and two isolates of a potentially new species that originated from Austrian foxes based on 93 biochemical reactions tested with the newly developed *Brucella *specific Micronaut™ microtiter plate. Hierarchical cluster analysis was performed by the Ward's linkage algorithm using the binary coded data based on the empirically set cut-off.

The panel of 93 discriminating reactions was re-evaluated for its usefulness in the identification of *Brucella *and the differentiation of its species and biovars using a broad spectrum of well characterized field strains. Both inter- and intra-assay variability were ascertained to be negligible. Results of the cluster analysis of the 113 strains investigated regarding their ability to metabolize the 93 selected substances supported our findings in the smaller collection of *Brucella *reference strains (Figure 3). Based on the metabolic profiles determined by the *Brucella *specific 96-well Micronaut™ plate, *B. melitensis *and *B. abortus *isolates fell into two distinct groups (Figure 3). *B. suis *(except for biovar 5) could be found in another group but the biovars 1, and 3 and 4 gathered together with *B. inopinata *and *B. canis *isolates, respectively. *B. suis *bv 2 could be separated by its substrate assimilation pattern. *B. suis *bv 5 showed metabolic traits similar to *B. ovis*, *B. neotomae *and the marine mammal strains. Each *Brucella *strain investigated revealed an individual metabolic profile.

Using the newly developed *Brucella *specific Micronaut™ biotyping assay, *B. abortus *bv 4, 5, and 7, *B. suis *bv 1-5, *B. ovis*, *B. neotomae*, *B. pinnipedialis*, *B. ceti*, *B. microti*, and *B. inopinata *could be discriminated within the genus with a specificity of 100% (Table 1). In contrast, members of the three *B. melitensis *biovars formed a homogenous group. Although the metabolic activity of *B. melitensis *strains did not correlate with the classical biotyping scheme, subgroups within the species could still be defined (Figure 3). Gram-negative microorganisms other than brucellae e.g. *Ochrobactrum intermedium*, *O. anthropi*, *Yersinia enterocolitica *O:9, and *Acinetobacter lwoffii *showed differing oxidative metabolic profiles and could clearly be distinguished from *Brucella *spp. in our experimental setting. Furthermore, a screening of the Micronaut-IDS database (Merlin Diagnostika) which is a widely used rapid identification system for Gram-negative and Gram-positive bacteria clearly discriminated brucellae from other bacterial taxa on the basis of four enzymatic reactions i.e. HP, Pyr-βNA (Pyr), urease, and NTA [Additional file 8, only clinically relevant bacteria are shown].

**Table 1:** Specificity of the *Brucella *specific Micronaut™ microtiter plate.

***Brucella *spp**.		Specificity in %			
**Species**	**Biovars**	**Biovar differentiation**		**Species differentiation**	

	**1**	0			
	**2**	75			
	**3**	90			
***B. abortus***	**4**	100		100	
	**5**	100			
	**6**	0			
	**7**	100			
	**9**	0			
			
	**1**	19		100	
***B. melitensis***	**2**	89			
	**3**	64			
			
	**1**	100	74	100	99
	**2**	100			
***B. suis***	**3**	100			
	**4**	100			
	**5**	100			
			
***B. ovis***				100	
***B. canis***				60	
***B. neotomae***				100	
***B. ceti***				100	
***B. pinnipedialis***				100	
***B. microti***				100	
***B. inopinata***				100	

Specificity of the Micronaut™ system to differentiate *Brucella *species and biovars.

The biotyping results were independent of the host and the geographic origin of *Brucella *isolates.

## Discussion

### Classical phenotyping and metabolic markers of *Brucella *spp

Although *Brucella *is a monophyletic genus, apparent differences between its species do exist e.g. host specificity and pathogenicity. Nowadays, *Brucella *species and biovars are distinguished by a limited number of microbiological tests measuring quantitative or qualitative differences of dye bacteriostasis, hydrogen sulfide production, urea hydrolysis, carbon dioxide requirement, bacteriophage sensitivity and agglutinin absorption. For at least half a century these microbiological procedures have not changed, although various new *Brucella *species showing variable phenotypic traits have been detected and new diagnostic methods have been developed.

Neither the classical biochemical tests nor antigenic properties and phage-sensitivity can be considered a reliable guide to the identification of *Brucella *species. Contradictory results were often reported [14]. However, variations in H_2_S production, CO_2 _requirement, a change in dye tolerance or atypical surface antigens i.e. inconsistent A and M antigens usually do not affect the oxidative metabolic pattern of a strain [15,16]. Metabolic activities have proven to be stable parameters allowing unambiguous species identification, particularly in strains which show conflicting identities by conventional determinative methods [14,17-19]. In addition, differing metabolism may help to describe new species [6,9,20]. In our series, two strains isolated from foxes in Austria (strain no. 110 and 111) which displayed an atypical metabolic pattern could be identified.

Oxidative metabolic profiles remain qualitatively stable for long periods of time and usually show no change in characteristic patterns after in vivo and in vitro passages [21]. However, quantitative differences in the oxidative metabolic rate of monosaccharides have been observed after multiple passages in vitro [21]. Variants in the oxidative metabolic pattern found among different CFUs of the same strain have been described in varying frequencies depending on *Brucella *species and biovars [22]. In our experiments, *B. suis *bv 1 showed the highest intra-strain variability in its enzymatic activity (*data not shown*).

Despite the stability of the metabolic markers and their consecutive usefulness in diagnostic assays, studies describing the differences in the metabolism of *Brucella *spp. have not been conducted for decades as the classical laboratory techniques are labour-intensive and very demanding. Especially Warburg manometry which is carried out in a respirometer measuring oxygen uptake has been widely used to determine oxidative metabolic patterns in order to describe and differentiate species, biovars, and atypical strains of the genus *Brucella*. Formerly, manometric studies on the metabolic activity of brucellae helped to quantitatively define the species classified within the genus [23]. However, due to the demanding techniques applied only a restricted number of strains and reactions were tested and various substrates e.g. D-asparagine, L-proline, adonitol, fructose and glucose were regarded as not useful for species and biovar differentiation [23,24]. In the comprehensive setting of this study most of these substrates also proved their usefulness.

Manometric studies have confirmed that a characteristic oxidative pattern for *Brucella *species exists whereas specific profiles for the biovars have not yet been described except for *B. suis *bv 1-4 [25]. Using the Micronaut™ system we were able to discriminate *B. abortus *bv 4, 5, and 7, *B. suis *bv 1-5, *B. ovis*, *B. neotomae*, *B. pinnipedialis*, *B. ceti*, *B. microti *and *B. inopinata *with a specificity of 100%. However, differentiation among the *B. melitensis *biovars was impossible as, according to their oxidative metabolic activity, they form a very homogenous group. The results of the cluster analysis based on our biotyping data (Figure 3) are in general concordance with the genotyping data acquired by Multiple Loci VNTR (Variable Number of Tandem Repeats) Analysis (MLVA) [26]. Neither biotyping nor genotyping proved a biovar specific clustering in *B. melitensis *strains [27]. Although we tested a substantial number of biochemical reactions we may have chosen the wrong set of substrates for the differentiation of *B. melitensis *strains, but the separation of this species in three biovars could also be somehow artificial.

### Biotyping of *Brucella *spp. using commercially available assays

If biological traits such as enzymatic activities are tested all potential variables must be reduced to a minimum to avoid intra- and inter-assay variations which may occur in addition to minimal biological variations. Commercial test systems offer a large number of quality controls both in the production chain and under experimental conditions.

Commercially available microtiter plates coated with various substrates to characterize the metabolic pattern of bacteria have already been used to describe new species of the genus *Brucella *e.g. the Biolog™ system for *B. ceti *[28] and the Micronaut™ system for *B. microti *and *B. inopinata *[6,9]. However, comprehensive metabolic studies including all currently known species and biovars are rare.

Using the Biolog™ GN MicroPlate system (Biolog, CA, USA) based on 44 differentially oxidized substrates, *B. melitensis*, *B. abortus *and *B. suis *isolates could be grouped into taxons identical with the presently recognized species [29]. However, only a restricted number of strains (n = 35) were tested and biovars were not differentiated. In a larger strain collection (n = 71) which included all biovars of the six classical *Brucella *species only 50% of the strains were correctly identified confirming the poor specificity of this commercially available, substrate mediated, tretrazolium identification technique [30]. López-Merino and colleagues used the Biotype 100™ carbon substrate assimilation system (bioMérieux, Marcy-L'Etoile, France) which comprises 99 carbohydrates, organic acids and other carbon substrates to discriminate *B. melitensis*, *B. abortus*, *B. suis *and *B. canis *[31]. Using the most discriminating carbon substrates i.e. D-glucose, D-trehalose, D-ribose, palatinose, L-fucose, L-malate, and DL-lactate more than 80% of the *B. melitensis *and *B. abortus *strains could be correctly identified. Similar to the *Brucella *specific Micronaut™ plate designed in this study *B. suis *and *B. canis *could not always be discriminated. The limited number of field isolates tested per species may have produced inconclusive results, particularly when only reference strains were available which are well known for atypical phenotypic traits. Future studies on larger strain collections may reveal more unique metabolic profiles suitable for species and biovar differentiation and also helpful to discriminate between *B. suis *bv 3 and *B. canis*. Nevertheless, the overall specificity for the identification of *Brucella *species using the Micronaut™ system reached 99%.

### Experimental conditions potentially interfering with bacterial metabolism and influencing biotyping results

Many experimental parameters may influence the metabolic activity of bacteria. For instance, oxidative rates may decrease if *Brucella *is prepared from 48 hours rather than 24 hours cultures [25] because *Brucella *is able to adapt to starvation. This effect does not seem to be important in the Micronaut™ system since turbidity is measured reflecting bacterial growth within a period of 48 hours as an indirect parameter for substrate utilization. Consequently, the bacteria have plenty of time to switch on all necessary metabolic pathways. Hence, the metabolic rate of glutamic acid may differ between *B. abortus *and *B. melitensis *[32] but after 48 h the substrate is entirely metabolized by both species. For the same reason *B. suis *has been described as inactive in the metabolism of glutamic acid but our results revealed extensive utilization of this substrate at least for the biovars 3-5 whereas the metabolization was variable in the biovars 1 and 2.

The growth medium can also have an effect on the utilization of substrates and brucellae may operate with alternate metabolic pathways leading to discrepant stimulatory effects in different assays [30]. Therefore, a minimal medium i.e. buffered sodium chloride peptone (from potatoes) solution was used in Taxa Profile™ and Micronaut™ plates to avoid interference with other potential substrates in the culture medium.

The rates of oxidation of various compounds are also strongly dependent on intact bacterial membranes and pH values [33,34]. In our experiments, asparagines were easily oxidized by most of the *Brucella *spp., but aspartic acid was not (exceptions were *B. suis *bv 4, *B. microti*, and *B. inopinata*). Furthermore, glutamic acid was oxidized, but intermediates in the pathway, such as α-ketoglutarate and succinate (except for *B. microti *and *B. inopinata*) were usually not. Lowering the pH of a reaction mixture containing intact cells of brucellae markedly increased the oxidation rate of these metabolites e.g. L-aspartate, α-ketoglutarate, succinate, fumarate, L-malate, oxaloacetate, pyruvate and acetate [34]. Differences between *Brucella *species may occur in the pH range at which the bacteria are able to utilize some of the substrates and therefore labile metabolic profiles can be observed [35]. Nevertheless, such reactions may be helpful for the differentiation of species and biovars if assay conditions are stable. The effect of extracellular adjustment of the pH upon intracellular enzymatic reactions can be explained by organic acids permeating the cell more readily when undissociated than when ionized. Hence, a pH change may overcome the permeability barrier for many substrates especially of the Krebs' cycle. For this reason our results do not easily reflect intracellular substrate utilization. In proteomic studies on intracellular brucellae and bacteria grown under stress conditions comparable to the intracellular niche of *Brucella*, enzymes of the TCA cycle i.e. the succinyl CoA synthetase and aconitate hydratase were found increased [36,37]. In contrast, intermediates of the TCA cycle such as citrate, isocitrate, α-ketoglutarate, succinate, malate, fumarate were not generally metabolized in vitro or showed variable metabolization in the different species such as oxaloacetic acid.

Although modelling of the intracellular niche of brucellae is not a topic of this study the Micronaut™ system might be helpful to investigate differences in the metabolic activity between the species under various growth conditions. This will allow a much deeper insight in the metabolic changes of intracellular compared to extracellular brucellae and will help to understand survival strategies of the pathogen under starvation, microaerobic and acidic conditions. In this context, a negative correlation between metabolic activity and the relative degree of virulence was observed among *B. abortus *strains [38]. Avirulent mutants of *B. melitensis*, *B. abortus *and *B. suis *that failed to replicate or survive in macrophages or animal models often had mutations in the carbohydrate metabolism [39]. In our study, *B. microti *which is not known to be human pathogenic was the metabolically most active species.

Independent of the method used a broad agreement can be observed for the utilization of carbohydrates by *Brucella *spp. whereas the results of the amino acid metabolism are more variable [3,16]. Differences in the oxidation rate of different isomers of the same amino acid have been described for short incubation periods, e.g. *B. suis *and *B. melitensis *are known to oxidize D-alanine more rapidly than the L-isomer [40] or *B. abortus *oxidized L-glutamic acid and L-asparagine rapidly whereas relatively slight activity was obtained with the D-isomers [38]. Differences in the metabolization rate could not be used for differentiation in our multi-substrate test. As many substrates were tested at the same time the incubation period was prolonged to 48 hours to ensure that each substrate was completely utilized. With a few exceptions, there are only minor differences in the general utilization of D- and L-isomers of amino acids within the same species [41]. Therefore both isomers of the same amino acid were only included three times in the Micronaut™ plate, i.e. D-/L-proline, D-/L-alanine, and D-/L-serine. In our experiments, opposing metabolic activity could be observed for the different isomers of proline in *B. abortus *bv 3, *B. suis *bv 2, and *B. canis*, for the isomers of alanine in *B. canis *and *B. neotomae*, and for the isomers of serine in *B. suis *bv 1, 2, and 4, *B. ovis*, *B. microti *and *B. inopinata*.

Further, substrate concentration may influence the metabolic activity of *Brucella *[34,38]. Although sample volumes are different in Taxa Profile™ and Micronaut™ plates the final substrate concentration is the same. Hence, apparently contradictory results in these two test systems which could be observed in our study cannot be explained by different concentrations of the same compound.

Because of the small volumes used in the Taxa Profile™ plate turbidity could not be measured due to technical limitations. Therefore the indicator phenol red was added to colorimetrically measure respiration. In contrast, in the 96-well Micronaut™ plate turbidity as a measure of bacterial growth was determined. The measurement of respiration instead of growth is much more sensitive since bacteria may respond metabolically by respiring but not by growing [42]. Hence, this effect may have led to differing results for the utilization of the same substrate on the two platforms. However, respiration could not be used in the genus *Brucella *since some strains are dependent on CO_2 _which catalyzes abiotic reduction of the dye.

As most metabolic pathways are encoded within the *Brucella *genome brucellae might present as fastidious due to slow growth. Although the genome sequence of *B. microti *is almost identical to that of *B. suis *with an overall sequence identity of 99.84% in aligned regions, phenotypically these species differ significantly which might be caused by variable gene regulations and different growth patterns [43].

Both respirometry and tetrazolium reduction assays proved that *B. abortus *is characteristically stimulated by L-alanine, L-asparagine and L-glutamate [30]. In contrast, the Micronaut™ results were heterogeneous for L-alanine in *B. abortus *strains. The differences in metabolic activity observed between these methods might be caused by the cut-off selected in our experiments. Deduced from the OD values measured with the Micronaut™ system three levels of substrate utilization could be defined: no/weak metabolic activity (-), moderate metabolic activity (+), and strong metabolic activity (++) [Additional file 7]. The different levels of oxidative metabolic activity on amino acid and carbohydrate substrates determined by Micronaut™ agreed with the oxygen uptake levels for most substrates measured by conventional manometric techniques [25]. However, owing to the dispersion of the individual OD values, quantitative differences are of limited practical relevance. The selection of cut-offs which delineated positive and negative metabolic activity greatly contributed to the clarification of the presentation of substrate utilization. Of course, the limit between two activity patterns is rather artificial.

## Conclusions

The results of the comprehensive biotyping study presented evidence that species of the genus *Brucella *can be correctly identified by their metabolic patterns. Although a range of metabolic properties allows clustering of *Brucella *into species and biovars clearly defined boundaries do not always exist.

Based on a selection of 93 different substrates out of 570 initially tested, a *Brucella *specific 96-well Micronaut™ microtiter plate was developed and successfully evaluated in a large panel of *Brucella *strains comprising all currently known species and biovars. Although the Micronaut™ system still requires a biological safety cabinet throughout the procedure it is much easier to handle and does not require the preparation of specific reagents leading to quicker results than conventional microbiological methods. Hence, the Micronaut™ system may replace or at least complement time-consuming tube testing. Furthermore, an easy to handle identification software facilitates its applicability for routine use.

The newly developed *Brucella *specific 96-well Micronaut™ plate fulfilled the performance criteria recommended for a typing assay, i.e. typeability, reproducibility, stability and discriminatory power. Although we were not able to examine the epidemiological concordance of our biotyping results, there are definite indications that the metabolic profiles of different isolates match within the same outbreak [16]. The Micronaut™ system has also proven to be invaluable in the characterization of otherwise untypable new species. However, reference and new strains should always be tested in the same series because the differences in oxidative metabolic profiles may not only be qualitative but also quantitative.

Biodiversity of *Brucella *spp. also reflects taxonomic (natural and evolutionary) relationships that exist between and among the organisms sequestered and clustered within the classification scheme. Hence, the Micronaut™ system is not only a diagnostic assay it can be a striking tool in functional taxonomy of the genus *Brucella*.

Our results may raise the question if the widely accepted biotyping scheme based on only a few phenotypic features is sufficient to get a clear idea of the true composition of the genus *Brucella *and will meet future demands. The new diagnostic approach presented in this study may help to overcome these limitations.

## Methods

### *Brucella *strains

*Brucella *spp. were characterized by classical microbiological methods according to Alton et al. (1988) [2]. Comprehensive biochemical phenotyping was performed on the *Brucella *reference strains representing all currently known species and their biovars as well as on up to 7 field isolates per species and biotype as far as available (Table 2). The consecutively established *Brucella *specific 96-well microtiter plate was evaluated testing the reference strains and a broad range of *Brucella *isolates (a total of 113 strains) originating from various animal hosts and human patients, i.e. *B. melitensis *bv 1 (n = 8), bv 2 (n = 14) and bv 3 (n = 11); *B. abortus *bv 1 (n = 9), bv 2 (n = 2), bv 3 (n = 5), bv 4 (n = 6), bv 5 (n = 1), bv 6 (n = 3), bv 7 (n = 1) and bv 9 (n = 3); *B. suis *bv 1 (n = 6), bv 2 (n = 8), bv 3 (n = 1), bv 4 (n = 2) and bv 5 (n = 1); *B. canis *(n = 5), *B. ovis *(n = 4), *B. neotomae *(n = 1), *B. pinnipedialis *(n = 8) and *B. ceti *(n = 1), *B. microti *(n = 10), *B. inopinata *(n = 1), and two atypical strains according to the hitherto existing biotyping scheme (Table 2). Isolates of diverse geographical origin were deliberately selected to gain a large variety of strains.

**Table 2:** *Brucella *strains tested for metabolic activity.

Species	Biovar	Strain	Culture collection	Host	Number of field isolates	
					
					Taxa Profile™ (570 substrates)	Micronaut™ *Brucella *plate (93 substrates)
	**1**	544	NCTC^a ^10093	Cattle	6	8
	**2**	86/8/59	NCTC 10501	Cattle	1	1
	**3**	Tulya	NCTC 10502	Human	4	4
***B. abortus***	**4**	292	NCTC 10503	Cattle	5	5
	**5**	B3196	NCTC 10504	Cattle	0	0
	**6**	870	NCTC 10505	Cattle	3	2
	7*	63175	NCTC 10506	Cattle	0	0
	**9**	C68	NCTC 10507	Cattle	2	2

	**1**	16 M	NCTC 10094	Goat	4	7
***B. melitensis***	**2**	63/9	NCTC 10508	Goat	5	13
	**3**	Ether	NCTC 10509	Goat	4	10

	**1**	1330	NCTC 10316	Swine	4	5
	**2**	Thomsen	NCTC 10510	Swine	6	7
***B. suis***	**3**	686	NCTC 10511	Swine	1	0
	**4**	40	AFSSA^b ^Ref. 40	Reindeer	1	1
	**5**	513	AFSSA Ref. 513	Wild rodent	0	0

***B. canis***		RM6/66	NCTC 10854	Dog	4	4
***B. ovis***		63/290	NCTC 10512	Sheep	2	3
***B. neotomae***		5K33	NCTC 10084	Desert rat	0	0
***B. pinnipedialis***			NCTC 12890	Common seal	7	7
***B. ceti***			NCTC 12891	Porpoise	0	0
***B. microti***			CCM^c ^4915	Common vole	1	9
***B. inopinata***		BO1	BCCN^d ^09-01	Human	0	0

***Unknown***			BfR^e ^11.1.001/002	Fox	0	2

**Total**		**23 reference strains**			**60 field isolates**	**90 field isolates**

*Brucella *reference strains and overview of field isolates tested with the Taxa Profile™ system and the newly developed *Brucella *specific Micronaut™ microtiter plate.

^a ^NCTC: National Collection of Type Cultures

^b ^AFSSA: Agence Française de Sécurité Sanitaire des Aliments

^c ^CCM: Czech Collection of Microorganisms

^d ^BCCN: *Brucella *Culture Collection from Nouzilly

^e ^BfR: Bundesinstitut für Risikobewertung

* The authenticity of the *B. abortus *bv 7 reference strain has been questioned; this strain remains as a potential reference strain until an agreement will be finally reached [44].

Various strains initially tested with the 384-well Taxa Profile™ plates were re-evaluated using the newly developed 96-well plate. In addition, a limited selection of closely related and clinically relevant bacteria was tested, i.e. *Acinetobacter lwoffii *(DSM 2403), *Yersinia enterocolitica *O:9 (IP-383 RKI/Paris), *Ochrobactrum intermedium *(CCUG 24964), *O. anthropi *(DSM 6882), *Enterococcus faecalis *(DSM 2570), *Escherichia coli *(DSM 1103), *Pseudomonas aeruginosa *(DSM 1117), and *Staphylococcus aureus *(DSM 2569).

### Culture and sample preparation

All strains were grown on *Brucella *agar for 48 h at 37°C with or without 10% CO_2 _depending on the needs of the particular species. Horse serum (10%) was added to the culture medium to facilitate the growth of *B. ovis*. Colony material was harvested and solubilised in 0.1% buffered sodium chloride peptone (from potatoes) solution and in sterile 0.9% NaCl for use in profile A or C plates and profile E plates, respectively. The turbidity of the bacterial suspension was adjusted to a 2.0 McFarland standard. Each well of the 384- and 96-well plates was inoculated with 25 μl and 100 μl of the respective preparation, respectively. The microtiter plates were incubated at 37°C for 48 h before reading.

### *Brucella *phenotyping

The metabolic activity of *Brucella *was comprehensively assessed using the Taxa Profile™ system (Merlin Diagnostika, Bornheim-Hersel, Germany) based on 384-well microtiter plates coated with various substrates. The Taxa Profile™ A microtiter plate allows testing of 191 different amines, amides, amino acids, other organic acids and heterocyclic and aromatic substrates [Additional file 1]. The Taxa Profile™ C microtiter plate enables the analysis of 191 different mono-, di-, tri- and polysaccharides and sugar derivates [Additional file 2]. Using the Taxa Profile™ E microtiter plate another 188 substrates to determine enzymatic activity were tested: 95 amino peptidase- and protease-reactions, 76 glycosidase-, phosphatase- and other esterase-reactions, and 17 classic reactions [Additional file 3]. According to manufacturer's instructions supplementary reagents were added to some wells of the Taxa Profile™ E microtiter plates to visualize substrate utilization. In addition, the indicator phenol red was added to all wells of the Taxa Profile™ A and C microtiter plates to optimize detection. The blank value was measured for each biochemical reaction on the same plate and subtracted from measured values. In order to assess inter-assay variability five independent experiments per strain were conducted.

For evaluation of the newly developed *Brucella *specific 96-well microtiter plate three trials per strain were run independently. Intra-assay variability was assessed with the reference strains testing all substances twice within the same experiment. Since the blank values measured on extra plates proved to be constant a fixed mean value of each substrate was subtracted from the measured data.

### Data acquisition and analysis

Turbidity and colour change were measured photometrically using a Multiskan Ascent^® ^photometer (Labsystems, Helsinki, Finland) at a wave length of 405 nm, 540 nm and 620 nm according to manufacturer's recommendations. Optimal OD cut-off values were empirically adapted from the preliminary test results of the 384-wells Taxa Profile™ microtiter plates.

Stable and discriminatory markers were selected to design a 96-well Micronaut™ plate (Figure 2) to identify bacteria of the genus *Brucella *and to classify their species and biovar.

Dendrograms were deduced from the biotyping data using SPSS version 12.0.2 (SPSS Inc., Chicago, IL, USA). First of all, three different character data sets were defined following the metabolic activity tested (Taxa Profile™ A ("amino acids"), C ("carbohydrates"), and E ("other enzymatic reactions")). Each character was considered as equal within the particular data set. Both the raw OD data and the binary coded data based on the empirically set cut-off were analyzed using the Pearson coefficient and the categorical coefficient, respectively. Hierarchical cluster analysis was performed by the Ward's linkage algorithm, and a dendrogram was generated. If necessary, analysis was repeated within each cluster for further discrimination. Secondly, a separate data analysis of the 23 *Brucella *reference strains representing the currently known species and biovars was performed including all biochemical reactions of the Taxa Profile™ system or exclusively the substrates selected for the newly developed plate. Finally, the whole collective of 113 strains tested with the *Brucella *specific Micronaut™ microtiter plate was analyzed to prove the diagnostic system. An identification table presenting quantitative and qualitative metabolic activity was created [Additional file 7] and the specificity of the test system to differentiate *Brucella *species and biovars was calculated (Table 1).

## Authors' contributions

SAD, HN, HT, KN, BA and AH were responsible for the study design. PB, CG, KN, SAD and HS were in charge of strain collection, selection and the biotyping work. SAD, PB and WK performed cluster analysis and checked the dataset for errors. KN, PB, SAD and HN designed the *Brucella *specific Micronaut™ microtiter plate. SAD wrote the report. KN, HN and WK helped to draft the manuscript. All authors read, commented and approved the final article.

## Supplementary Material

Additional file 1 **List of biochemical reactions tested with the Taxa Profile™ A plate**. The Taxa Profile™ A microtiter plate allows testing of 191 different amines, amides, amino acids, other organic acids and heterocyclic and aromatic substrates. Click here for file

Additional file 2 **List of biochemical reactions tested with the Taxa Profile™ C plate**. The Taxa Profile™ C microtiter plate enables the analysis of 191 different mono-, di-, tri- and polysaccharides and sugar derivates. Click here for file

Additional file 3 **List of biochemical reactions tested with the Taxa Profile™ E plate**. The Taxa Profile™ E microtiter plate is configured to determine the enzymatic activity of 95 amino peptidases and proteases, 76 glycosidases, phosphatases and other esterases, and also includes 17 classic reactions. Click here for file

Additional file 4 **Cluster analysis of *Brucella *reference and field strains based on their amino acid metabolism**. Cluster analysis of 83 *Brucella *and 2 *Ochrobactrum *strains based on 191 biochemical reactions tested with the Taxa Profile™ A plate. Hierarchical cluster analysis was performed by the Ward's linkage algorithm using the raw OD data. Click here for file

Additional file 5 **Cluster analysis of *Brucella *reference and field strains based on their carbohydrate metabolism**. Cluster analysis of 83 *Brucella *and 2 *Ochrobactrum *strains based on 191 biochemical reactions tested with the Taxa Profile™ C plate. Hierarchical cluster analysis was performed by the Ward's linkage algorithm using the raw OD data. Click here for file

Additional file 6 **Cluster analysis of *Brucella *reference and field strains based on specific enzymatic reactions**. Cluster analysis of 83 *Brucella *and 2 *Ochrobactrum *strains based on 188 biochemical reactions tested with the Taxa Profile™ E plate. Hierarchical cluster analysis was performed by the Ward's linkage algorithm using the raw OD data. Click here for file

Additional file 7 **Metabolic activity of *Brucella *strains**. Relative frequency (%) of positive and negative metabolic activity among 23 *Brucella *reference strains and 90 field isolates (Table 2) observed for the 93 substances tested in the *Brucella *specific Micronaut™ plate. Both quality and relative quantity are presented: - no metabolic activity (highlighted in green), + moderate metabolic activity (in orange), ++ strong metabolic activity (in red). Click here for file

Additional file 8 **Separation of *Brucella *spp. from clinically relevant bacteria**. Relative frequency (%) of positive metabolic activity among *Brucella *and other bacteria observed for HP, Pyr-βNA (Pyr), urease, and NTA. Four enzymatic reactions which were revealed by the Micronaut-IDS database screening clearly discriminated *Brucella *from clinically relevant bacteria of other genera. Click here for file
