# Supplementary material for: Differential phenotyping of Brucella species using a newly developed semi-automated metabolic system
Source: BMC Microbiol. 2010 Oct 23;10:269. doi: 10.1186/1471-2180-10-269 (PMC2984481; doi:10.1186/1471-2180-10-269)
Supplement: Additional file 1 — List of biochemical reactions tested with the Taxa Profile™ A plate. The Taxa Profile™ A microtiter plate allows testing of 191 different amines, amides, amino acids, other organic acids and heterocyclic and aromatic substrates. [file 1471-2180-10-269-S1.PDF]

Katalog-Nr.	Kategorie	Bezeichnung	**M E R L I N** Diagnostika	Aktualisierungsdatum: **01.01.1990**
EF-118-001	M	MICRONAUT- PROFILE A		Druckdatum: **03.11.2004**
**Layout:** 2 Tests	K			

	1	2	3	4	5	6	7	8	9	10	11	12	13	14	15	16	17	18	19	20	21	22	23	24
A	Gly	L-Ile	L-Met	L-Tyr	L-Glu	L-NVal	AcLeu	tHPro	L-Cit	Putre	DABut	LTyrA	aAAdi	Acet	Propn	Mali	Citrc	Capra	Suber	Oleic	Orota	Catec	Anthr	Coum
B	ß-Ala	D-Ile	D-Met	D-Tyr	D-Glu	D-NVal	AcArg	L-Cyss	L-Caro	Sperm	ßABut	pACin	ACapr	Thio	dlLac	yHBut	Itaco	hiCap	Dipic	Doxyc	Xanth	Benzo	3aBen	GuamP
C	L-Ala	L-Pro	L-Ser	L-Asn	L-Lys	l-NLeu	AcGlu	L-Cysa	L-Carn	Sperd	gABut	AcetA	Trigo	Glyc	Fuma	ßHBut	Mesac	Picol	oCoum	Imida	Hypox	hBenz	Phaa	Guano
D	D-Ala	D-Pro	D-Ser	D-Asn	D-Lys	d-NLeu	AcAsp	LHCyss	D-Carn	HipArg	yGBut	Betai	Tyram	Glyx	Male	iBut	Levul	iCita	pCoum	Succi	Adeni	Salic	DMand	Thiam
E	L-Val	L-Phe	L-Thr	L-Gln	l-Arg	l-HSer	CLeu	L-Pyrg	diAPim	AlaGln	5ALec	Sarc	dlKyn	Oxal	Succa	aHiBu	aKiVal	Cita	Sebac	Urac	Thymi	aBenz	DlMan	Catei
F	D-Val	D-Phe	D-Thr	D-Gln	D-Arg	d-HSer	DCSer	D-Pyrg	APim	AlaGly	LAlaA	Allat	KynA	Propl	Oxala	iVala	akVala	Sorba	Hepta	Barba	Nicot	mhBen	iVana	Folic
G	L-Leu	L-Trp	L-Cys	L-Asp	L-His	l-HArg	Hip	L-Orn	Creat	Gluth	LLeuA	GuaA	Taur	Malo	DTart	nVala	Gluta	Shiki	Azel	Cytos	NiCat	ProCa	Vana	Tauro
H	D-Leu	D-Trp	D-Cys	D-Asp	D-His	dlHCys	pAHip	D-Orn	Cadav	LABut	LProA	GuaSu	Etha	Pyru	Crot	aKeto	Adipa	Pimel	Capri	Crtin	Niaci	Galli	3aaPh	ACO
I	Gly	L-Ile	L-Met	L-Tyr	L-Glu	L-NVal	AcLeu	tHPro	L-Cit	Putre	DABut	LTyrA	aAAdi	Acet	Propn	Mali	Citrc	Capra	Suber	Oleic	Orota	Catec	Anthr	Coum
J	ß-Ala	D-Ile	D-Met	D-Tyr	D-Glu	D-NVal	AcArg	L-Cyss	L-Caro	Sperm	ßABut	pACin	ACapr	Thio	dlLac	yHBut	Itaco	hiCap	Dipic	Doxyc	Xanth	Benzo	3aBen	GuamP
K	L-Ala	L-Pro	L-Ser	L-Asn	L-Lys	l-NLeu	AcGlu	L-Cysa	L-Carn	Sperd	gABut	AcetA	Trigo	Glyc	Fuma	ßHBut	Mesac	Picol	oCoum	Imida	Hypox	hBenz	Phaa	Guano
L	D-Ala	D-Pro	D-Ser	D-Asn	D-Lys	d-NLeu	AcAsp	LHCyss	D-Carn	HipArg	yGBut	Betai	Tyram	Glyx	Male	iBut	Levul	iCita	pCoum	Succi	Adeni	Salic	DMand	Thiam
M	L-Val	L-Phe	L-Thr	L-Gln	l-Arg	l-HSer	CLeu	L-Pyrg	diAPim	AlaGln	5ALec	Sarc	dlKyn	Oxal	Succa	aHiBu	aKiVal	Cita	Sebac	Urac	Thymi	aBenz	DlMan	Catei
N	D-Val	D-Phe	D-Thr	D-Gln	D-Arg	d-HSer	DCSer	D-Pyrg	APim	AlaGly	LAlaA	Allat	KynA	Propl	Oxala	iVala	akVala	Sorba	Hepta	Barba	Nicot	mhBen	iVana	Folic
O	L-Leu	L-Trp	L-Cys	L-Asp	L-His	l-HArg	Hip	L-Orn	Creat	Gluth	LLeuA	GuaA	Taur	Malo	DTart	nVala	Gluta	Shiki	Azel	Cytos	NiCat	ProCa	Vana	Tauro
P	D-Leu	D-Trp	D-Cys	D-Asp	D-His	dlHCys	pAHip	D-Orn	Cadav	LABut	LProA	GuaSu	Etha	Pyru	Crot	aKeto	Adipa	Pimel	Capri	Crtin	Niaci	Galli	3aaPh	ACO

[Angabe der Konzentrationen in µg/ml]

L E G E N D E

[Ausgabe von PPS-Kürzeln]

(+)-catechin	Catei	Dl-mandelic acid	DlMan	a-ketoglutaric acid	aKeto	dl-lactic acid	dlLac	maleic acid	Male	suberic acid	Suber
(-)-shikimic acid	Shiki	L-a-amino-n-butyric	LABut	a-ketoisovaleric ac.	aKiVal	dl-ß-amino-n-butyric	ßABut	malonic acid	Malo	succinic acid	Succa
2-amino-1-methyl-	Crtin	acid		a-ketovaleric acid	akVala	acid		mesaconic acid	Mesac	succinimid	Succi
2-imidazolin-4-one		L-alanin	L-Ala	acetamid	AcetA	dl-ß-hxdroxybutyric	ßHBut	n-acetyl-dl-glutamic	AcGlu	taurin	Taur
3-acetamidophenol	3aaPh	L-alaninamid HCl	LAlaA	adenin	Adeni	acid		acid		taurocholic acid	Tauro
3-aminobenzamid	3aBen	L-arginin HCl	l-Arg	adipic acid	Adipa	ethanolamin	Etha	n-acetyl-l-aspartic	AcAsp	thiamin HCl	Thiam
4-nitrocatechol	NiCat	L-asparagin	L-Asn	ala-gln	AlaGln	folic acid	Folic	acid		thioglycolic acid	Thio
5-aminolevulinic ac.	5ALec	L-aspartic-acid	L-Asp	ala-gly	AlaGly	fumaric acid	Fuma	n-acetyl-l-leucin	AcLeu	thymin	Thymi
Assimilations Kontr.	ACO	L-carnitin HCl	L-Carn	allantoin	Allat	g-amino-n-butyric ac	gABut	n-caproic acid	Capra	trans-	tHPro
D(-)mandelic acid	DMand	L-carnosin	L-Caro	anthralinic acid	Anthr	gallic acid	Galli	n-valeric acid	nVala	4-hxdroxy-l-prolin	
D(-)tartaric acid	DTart	L-citrullin	L-Cit	azelaic acid	Azel	glutaric acid	Gluta	na-acetyl-l-arginin	AcArg	trigonellin	Trigo
D-His	D-His	L-cysteic acid	L-Cysa	barbituric acid	Barba	glutathion reduced	Gluth	niacinamid	Niaci	tyramin HCl	Tyram
D-a-aminobutyric	DABut	L-cystein	L-Cys	benzoic acid	Benzo	from free acid		nicotinic acid	Nicot	uracil	Urac
aci.		L-cystin	L-Cyss	betain	Betai	glycin free base	Gly	o-coumaric acid	oCoum	vanillic acid	Vana
D-alanin	D-Ala	L-glutamic acid	L-Glu	cadaverin	Cadav	glycolic acid	Glyc	oleic acid	Oleic	xanthin	Xanth
D-arginin HCl	D-Arg	L-glutamin	L-Gln	capric acid	Capri	glyoxylic acid	Glyx	orotic acid	Orota	y-guanidinobutyric	yGBut
D-asparagin	D-Asn	L-histidin HCl	L-His	catechol	Catec	guanidinosuccinic ac	GuaSu	oxalacetic acid	Oxala	acid	
D-aspartic acid	D-Asp	L-homocyctin	LHCyss	citraconic acid	Citrc	guanidoacetic acid	GuaA	oxalic acid	Oxal	y-hydroxybutyric ac.	yHBut
D-carnitin	D-Carn	L-isoleucin	L-Ile	citric acid	Cita	guanosin	Guano	p-aminobenzoic acid	aBenz	ß-alanin	ß-Ala
D-cycloserin from	DCSer	L-leucin	L-Leu	coumarin	Coum	guanosin	GuamP	p-aminocinnamic acid	pACin		
microbial source		L-leucinamid HCl	LLeuA	creatin hydrat	Creat	2´-monophosphat		p-aminohippuric acid	pAHip		
D-cystein	D-Cys	L-lysin 2*HCl	L-Lys	crotonic acid	Crot	heptanoic acid	Hepta	p-coumaric acid	pCoum		
D-glutamic acid	D-Glu	L-methionin	L-Met	cycloleucin	CLeu	hippuric acid	Hip	p-hydroxybenzoic ac.	hBenz		
D-glutamin	D-Gln	L-norvalin	L-NVal	cytosin	Cytos	hippuryl-arg	HipArg	phenylacetic acid	Phaa		
D-isoleucin	D-Ile	L-ornithin HCl	L-Orn	d(+)malic acid	Mali	hypoxanthin	Hypox	picolinic acid	Picol		
D-leucin	D-Leu	L-phenylalanin	L-Phe	d-homoserin	d-HSer	imidazol HCl	Imida	pimelic acid	Pimel		
D-lysin	D-Lys	L-prolin	L-Pro	d-norleucin	d-NLeu	isobutyric acid	iBut	propiolic acid	Propl		
D-methionin	D-Met	L-prolinamid HCl	LProA	deoxycholic acid	Doxyc	isovaleric acid	iVala	propionic acid	Propn		
D-norvalin	D-NVal	L-pyroglutamic acid	L-Pyrg	dipicolinic acid	Dipic	isovanillic acid	iVana	protocatechuic acid	ProCa		
D-ornithin HCl	D-Orn	L-serin	L-Ser	dl-a,e-diaminopimelic	diAPim	itaconic acid	Itaco	putrescin 2HCl	Putre		
D-phenylalanin	D-Phe	L-threonin	L-Thr	acid		kynurenic acid	KynA	pyruvic acid	Pyru		
D-prolin	D-Pro	L-tryptophan	L-Trp	dl-a-	ACapr	l-a-hydroxyisocaproic	hiCap	salicylic acid	Salic		
D-pyroglutamic acid	D-Pyrg	L-tyrosin free base	L-Tyr	amino-e-caprolactam		acid		sarcosin HCl	Sarc		
D-serin	D-Ser	L-tyrosinamid	LTyrA	dl-a-aminoadipic ac.	aAAdi	l-homoarginin HCl	l-HArg	sebacic acid	Sebac		
D-threonin	D-Thr	free base		dl-a-aminopimelic ac	APim	l-homoserin	l-HSer	sodium acetat	Acet		
D-tryptophan A	D-Trp	L-valin	L-Val	dl-homocystein	dlHCys	l-norleucin	l-NLeu	sorbic acid	Sorba		
D-tyrosin	D-Tyr	a-hxdroxyisobutyric	aHiBu	dl-isocitric acid	dlKyn	levulinic acid	Levul	spermidin 3*HCl	Sperd		
D-valin	D-Val	acid		dl-kynurenin	dlKyn	m-hydrobenzoic acid	mhBen	spermin 5*HCl	Sperm		
