# Supplementary material for: Differential phenotyping of Brucella species using a newly developed semi-automated metabolic system
Source: BMC Microbiol. 2010 Oct 23;10:269. doi: 10.1186/1471-2180-10-269 (PMC2984481; doi:10.1186/1471-2180-10-269)
Supplement: Additional file 2 — List of biochemical reactions tested with the Taxa Profile™ C plate. The Taxa Profile™ C microtiter plate enables the analysis of 191 different mono-, di-, tri- and polysaccharides and sugar derivates. [file 1471-2180-10-269-S2.PDF]

Katalog-Nr.	Kategorie	Bezeichnung	MERLIN Diagnostika	Aktualisierungsdatum:
EF-117-001	M	MICRONAUT- PROFILE C		01.01.1990
Layout: 2 Tests	K			Druckdatum: 03.11.2004

	1	2	3	4	5	6	7	8	9	10	11	12	13	14	15	16	17	18	19	20	21	22	23	24
A	GLYOL	D-ARA	D-XYL	LYXA	D-FRU	L-TAL	FUCOL	FRUGP	MaRHA	GLUCY	SAAS	GULOL	GLA	GLUHS	MALTO	TREH	HQGLU	SAL	MALOL	a-CYD	GUMXA	CMAMY	HESTA	PEC
B	aGLYP	L-ARA	L-AOL	MdXYL	D-GAL	D-TAL	DULOL	DOGAL	MßGAL	GALAS	GALS	GALOL	MANA	MGLCA	MATS	CELOB	NßGLU	IPGF	LABIS	ß-CYD	GUMA	CMCEL	HMCEL	ARBS
C	ERO	L-LYX	D-AOL	MßARA	D-GLU	D-TAG	IDOL	DOGLU	MßGLU	GLOS	ASBS	GLUSL	AGALA	CYTID	ß-LAC	TURA	FRAX	IPAF	MEBIS	y-CYD	GEGUM	DEX	INULD	STA
D	ERU	D-LYX	ADON	MßLAR	L-MAN	DIGTO	MANOL	DIRIH	MEGAL	PGCHA	MURS	MANSL	AMANA	INOP	a-LAC	MLZT	MIPM	IPFP	BGLA	Hß-CYD	MUCIN	FCLL	LICH	XYN
E	THREO	RIBU	XYTOL	MßXYL	D-MAN	INOL	SOROL	L-FUC	MaGAL	ASCOS	PANTI	GLOSL	AGLA	INON	LACTU	MALTR	IPXF	IPGP	GLAPA	DMCYD	PULL	FUCD	LEV	COLMS
F	EROL	L-RIB	RIBOP	CHLA	D-PSI	DNSOL	AHGAL	D-FUC	MEGLU	2KGS	GALL	SAS1L	SEDHU	XANTH	MELI	THGAL	GALAR	IPSF	PAN	TMCYD	STACH	GLYCO	MGCHI	PGALS
G	L-TOL	D-RIB	RIBOS	IPRAS	L-SOR	DNMOL	AHMAN	MaMAN	MaGLU	5KGS	GULL	SAS3L	GLUHO	BGLOL	PAL	AMY	ARBU	IPMF	RAFI	GUMTA	AMPEC	ßGLUC	MGXYN	HYLS
H	D-TOL	L-XYL	DORIB	D-ALL	D-SOR	DNAOL	AHGLU	L-RHA	PGF	GLUS	GLYHL	GALA	MANHO	ß-GEN	SUCR	NAR	AßGAL	IPMOL	ARGAL	GUMKA	CHI	GCCHI	MECEL	ACO
I	GLYOL	D-ARA	D-XYL	LYXA	D-FRU	L-TAL	FUCOL	FRUGP	MaRHA	GLUCY	SAAS	GULOL	GLA	GLUHS	MALTO	TREH	HQGLU	SAL	MALOL	a-CYD	GUMXA	CMAMY	HESTA	PEC
J	aGLYP	L-ARA	L-AOL	MdXYL	D-GAL	D-TAL	DULOL	DOGAL	MßGAL	GALAS	GALS	GALOL	MANA	MGLCA	MATS	CELOB	NßGLU	IPGF	LABIS	ß-CYD	GUMA	CMCEL	HMCEL	ARBS
K	ERO	L-LYX	D-AOL	MßARA	D-GLU	D-TAG	IDOL	DOGLU	MßGLU	GLOS	ASBS	GLUSL	AGALA	CYTID	ß-LAC	TURA	FRAX	IPAF	MEBIS	y-CYD	GEGUM	DEX	INULD	STA
L	ERU	D-LYX	ADON	MßLAR	L-MAN	DIGTO	MANOL	DIRIH	MEGAL	PGCHA	MURS	MANSL	AMANA	INOP	a-LAC	MLZT	MIPM	IPFP	BGLA	Hß-CYD	MUCIN	FCLL	LICH	XYN
M	THREO	RIBU	XYTOL	MßXYL	D-MAN	INOL	SOROL	L-FUC	MaGAL	ASCOS	PANTI	GLOSL	AGLA	INON	LACTU	MALTR	IPXF	IPGP	GLAPA	DMCYD	PULL	FUCD	LEV	COLMS
N	EROL	L-RIB	RIBOP	CHLA	D-PSI	DNSOL	AHGAL	D-FUC	MEGLU	2KGS	GALL	SAS1L	SEDHU	XANTH	MELI	THGAL	GALAR	IPSF	PAN	TMCYD	STACH	GLYCO	MGCHI	PGALS
O	L-TOL	D-RIB	RIBOS	IPRAS	L-SOR	DNMOL	AHMAN	MaMAN	MaGLU	5KGS	GULL	SAS3L	GLUHO	BGLOL	PAL	AMY	ARBU	IPMF	RAFI	GUMTA	AMPEC	ßGLUC	MGXYN	HYLS
P	D-TOL	L-XYL	DORIB	D-ALL	D-SOR	DNAOL	AHGLU	L-RHA	PGF	GLUS	GLYHL	GALA	MANHO	ß-GEN	SUCR	NAR	AßGAL	IPMOL	ARGAL	GUMKA	CHI	GCCHI	MECEL	ACO

[Angabe der Konzentrationen in µg/ml]

LEGENDE

[Ausgabe von PPS-Kürzeln]

(s)-(-)-Glycidol	GLYOL	D-arabinose		D(-)-Ribose	D-RIB	Gellan Gum (Gelrite)	GEGU	L-Threitol	L-TOL	Xanthosin	XANTH
1,2-o-Isopropylidene-	PGF	4-o-Methyl-	MGXYN	D-Amygdalin	AMY	Glycogen Typ VII	GLYCO	Lactobionic acid	LABIS	Xylan from birchwood	XYN
a-D-glucofuran		D-glucurono-D-xylan		D-Fruct.-6-phosphat	FRUGP	Glycol chitosan	GCCHI	Lactulose	LACTU	Xylitol	XYTOL
1,2:3,4-di-o-Isopr.idene-	IPGP	5-Keto-D-gluton.acid	5KGS	D-Galacton.a.-y-lac.	GALL	Gum Arabic	GUMA	Levan	LEV	a-Chloralose-ß-anom.	CHLA
D-galactopyr.		6-Phosphoglucon.acid	PGCHA	D-Galactonic acid	GALAS	Gum karaya	GUMKA	Lichenan	LICH	a-Cyclodextrin	a-CYD
1,2:3,5-di-o-Isopropylide	IPXF	6-o-Meth.D-galactop.	MEGAL	D-Galacturonic acid	GALS	Gum tragacanth	GUMTA	Maltitol	MALOL	a-D-Glucohepton.acid	GLUHS
D-xylofuran.		8-Hydroxyquino.ß-	HQGLU	D-Gluc.-L-cystein	GLUCY	Gum xanthan	GUMXA	Maltose grade I	MATS	a-D-Talose	D-TAL
1,2:5,6-di-o-Isopr.idene-	IPGF	D-glucopyranosid		D-Glucoheptose	GLUHO	Hep.(2,3,6-tri-o-meth.)-	TMCYD	Maltotriose	MALTR	a-L-Rhamnose	L-RHA
a-D-glucofur.		Adonit	ADON	D-Gluconic a. lacton	GLUSL	ß-cyclodextrin		Melibionic acid	MEBIS	a-Lactose	a-LAC
1,2:5,6-di-o-Isopr.idene.-	IPAF	Amylopectin f.potato	AMPEC	D-Gluconic acid	GLOS	Hept.(2,6-di-o-meth.)-	DMCYD	Melibiose	MELI	i-Erythriol	EROL
a-D-allofuran.		Arabic acid	ARBS	D-Glucuronic a.lac.	GLOSL	ß-cyclodextrin		Methyl 2,3-o-isopr.i	MIPM	iso-Maltose	MALTO
1,2:5,6-di-o-Isopr.idene.-	IPFP	Arabinogalactan	ARGAL	D-Glucuronic acid	GLUS	Hyaluronic acid	HYLS	dene-a-D-mannopyr.		myo-Inosit	INOL
ß-D-fructop.		Arbutin	ARBU	D-Glycohepton-1,4-l.	GLYHL	Hydroxyethyl starch	HESTA	Methyl-D-xylopyran.	MdXYL	n-Acetyl-D-galactos.	AGALA
1,2:5,6-di-o-Isopropylide	IPMOL	Assimilations Kontr.	ACO	D-Gulono-y-lacton	GULL	Hydroxypropyl-	Hß-CY	Methyl-a-D-galactop.	MaGAL	n-Acetyl-D-glucos.	AGLA
D-mannitol		Carboxymethylamylose	CMAMY	D-Isoascorbic acid	ASBS	ß-cyclodextrin		Methyl-a-D-glucop.	MaGLU	n-Acetyl-D-mannosam.	AMANA
1,6-Anh.-ß-D-mannop.	AHMAN	Carboxymetylcellul.	CMCEL	D-Lyxose	D-LYX	Hydroxypropylmethlce	HMCEL	Methyl-a-D-mannop.	MaMAN	n-BenzoylD-glucosam.	BGLA
1,6-Anh.-ß-galactop.	AHGAL	Cellobiose	CELOB	D-Mannitol	MANOL	llulose		Methyl-a-L-rhamnop.	MaRHA	n-Methyl-D-glucamin	MGLCA
1,6-Anhyd.-ß-D-gluc.	AHGLU	Chitosan	CHI	D-Mannoheptose	MANHO	Inosin	INON	Methyl-ß-D-arabinop.	MßARA	n-Nonyl-ß-D-glucop.	NßGLU
1-Deoxy-	DNAOL	Colominic Acid	COLMS	D-Mannosamin	MANA	Inosin	INOP	Methyl-ß-D-galactop.	MßGAL	p-Aminoph.-ß-D-gal.	AßGAL
1-nitro-D-altriotol		Cytidin	CYTID	D-Psicose	D-PSI	5`-monophosphat		Methyl-ß-D-glucop.	MßGLU	y-Cyclodextrin	y-CYD
1-Deoxy-	DNMOL	D(+)-Arabitol	D-AOL	D-Rib.-5-phosphat	RIBOP	Inulin f.dahlia tub.	INULD	Methyl-ß-D-xylop.	MßXYL	ß-Cyclodextrin	ß-CYD
1-nitro-D-mannitol		D(+)-Digitose	DIGTO	D-Ribonic acid	RIBOS	L(+)-Arabinose	L-ARA	Methyl-ß-L-arabinop.	MßLAR	ß-D-Allose	D-ALL
1-Deoxy-	DNSOL	D(+)-Fucose	D-FUC	D-Ribulose	RIBU	L(+)-Ribose	L-RIB	Methylcellulose	MECEL	ß-D-Glucosa.pentaac.	GLAPA
1-nitro-D-sorbitol		D(+)-Galactosamin	GALA	D-Saccharic acid	SAAS	L(+)-Threose	THREO	Methylglycolchitosan	MGCHI	ß-Gentiobiose	ß-GEN
1-Thio-ß-D-galactop.	THGAL	D(+)-Galactose	D-GAL	D-Saccharic	SAS1L	L(-)-Arabitol	L-AOL	Mucin Typ III	MUCIN	ß-Glucan	ßGLUC
2,3-o-Isopr.id.-ß-D-ribof.s	IPRAS	D(+)-Glucosamin	GLA	acid 1,4-lacton		L(-)-Fucose	L-FUC	Muramic acid	MURS	ß-Lactose	ß-LAC
p-toluens.		D(+)-Glucose	D-GLU	D-Saccharic	SAS3L	L(-)-Mannose	L-MAN	Naringin	NAR		
2,3:4,6-di-o-Isopr.idene-	IPSF	D(+)-Mannose	D-MAN	acid 3,6-lacton		L(-)-Sorbose	L-SOR	Palatinose	PAL		
a-L-sorbofuran.		D(+)-Melezitose	MLZT	D-Sorbitol	SOROL	L(-)-Talose	L-TAL	Panose	PAN		
2,3:5,6-di-o-Isopr.idene-	IPMF	D(+)-Raffinose	RAFI	D-Tagatose	D-TAG	L(-)-Xylose	L-XYL	Pectin from apple	PEC		
a-D-mannofuran.		D(+)-Sorbose	D-SOR	D-Threitol	D-TOL	L-Ascorbic acid	ASCOS	Polygalacturon.acid	PGALS		
2-Deoxy-D-galactose	DOGAL	D(+)-Trehalose	TREH	DL-a-Glycerophosphat	aGLYP	L-Erythrulose	ERU	Pullulan	PULL		
2-Deoxy-D-glucose	DOGLU	D(+)-Xylose	D-XYL	DL-a-o-Benzylglycer.	BGLO	L-Fucitol	FUCOL	SAL	SAL		
2-Deoxy-D-ribohexop.	DIRIH	D(-)-Arabinose	D-ARA	Dextran	DEX	L-Galacton.a.y-lac.	GALOL	Sedoheptulose	SEDHU		
2-Deoxy-D-ribose	DORIB	D(-)-Erytrhose	ERO	Dulcitol	DULOL	L-Gulono-y-lacton	GULOL	Stachyose	STACH		
2-Keto-D-glucon.acid	2KGS	D(-)-Fructose	D-FRU	Ficoll Typ 70	FCLL	L-Iditol	IDOL	Strach soluble	STA		
3-o-Methyl-D-glucop.	MEGLU	D(-)-Lyxosylamin	LYXA	Fraxin	FUCD	L-Lyxose	L-LYX	Sucrose	SUCR		
3-o-ß-D-Galactopyranosy	GALAR	D(-)-Pantoyllaceton	PANTI	Fucoidan	FUCD	L-Mannoic a. -lacton	MANSL	Turanose	TURA		
