# Supplementary material for: Differential phenotyping of Brucella species using a newly developed semi-automated metabolic system
Source: BMC Microbiol. 2010 Oct 23;10:269. doi: 10.1186/1471-2180-10-269 (PMC2984481; doi:10.1186/1471-2180-10-269)
Supplement: Additional file 3 — List of biochemical reactions tested with the Taxa Profile™ E plate. The Taxa Profile™ E microtiter plate is configured to determine the enzymatic activity of 95 amino peptidases and proteases, 76 glycosidases, phosphatases and other esterases, and also includes 17 classic reactions. [file 1471-2180-10-269-S3.PDF]

Katalog-Nr.	Kategorie	Bezeichnung	**M E R L I N** Diagnostika	Aktualisierungsdatum: **01.01.1990** Druckdatum: **03.11.2004**
EF-119-001	M	MICRONAUT- PROFILE E		
**Layout:** 2 Tests	K			

	1	2	3	4	5	6	7	8	9	10	11	12	13	14	15	16	17	18	19	20	21	22	23	24
A	G	I	T	K	CIT	AcGK	SY	AcKA	AAA	PyHBP	GNA	DNA	aARA7	oaGAL7	ßIFUC7	nGLUA7	ßLAC7	INDS7	PNPX4	aCHIT4	ßARA5	nßGAA5	ßMAN5	AST
B	dA	P	C	R	GG	AA	DA	AcKR	VTS	ALYL	LANA	ENAOH	ßARA7	tßGAL7	ßdFUC7	aMAN7	aAMY7	CATS7	PNPG4	aMAN4	PNPX5	aGLU5	PGUR5	HIP
C	IA	HP	Y	BzdIR	GA	dAdA	DK	HS	PKA	APAR	DANA	KNA	aARAF7	2vßGAL	ßtFUC7	ßMAN7	t5MP7	oACET7	ßdFUC4	PGUR4	PNPG5	ßGLU5	aRHA5	IND
D	ßA	F	Q	BzR	GP	AR	DR	GPL	FPA	AFPA	VNA	RNA	PNPX7	GAL6P7	aGLU7	PGUR7	DIP7	pACET7	nßGAA4	ßCEL4	aGAL5	aCHIT5	ßCEL5	NTA
E	V	GyF	N	H	GF	LG	GyDK	GyGGF	sFGL	AcAAPA	LNA	BzRNA	ONPX7	nßGAA7	ßGLU7	aRHA7	PHOS7	BUT7	aGLU4	PHOS4	oaGAL5	CHIT5	aMAL5	NTI
F	V4M	W	D	Orn	GdF	PR	EE	AAF	sYGL	LLVYS	ENA	PYRNA	aXYL7	naGAA7	tßGLU7	ßCEL7	BISPH7	PHOS8	ßGLU4	aARA5	ßdFUC5	aMAN5	PHOS5	PCA
G	IL	M	E	Pyr	GW	FP	EH	APA	HLH	G4DK	PNA	GPNA	PNPG7	onGAA7	aCHIT7	aMAL7	PHCHO7	TTR	ESC	DECO	ADH	LDC	UREI	PCC
H	dL	S	EßNa	Hyp	GR	FR	RR	AFP	PyHP	PepK	FNA	AFPNA	aGAL7	aIFUC7	CHIT7	ßMAL7	SULF7	VP	H2S	H2S/ESCO	GDC	ODC	UREII	TDA
I	G	I	T	K	CIT	AcGK	SY	AcKA	AAA	PyHBP	GNA	DNA	aARA7	oaGAL7	ßIFUC7	nGLUA7	ßLAC7	INDS7	PNPX4	aCHIT4	ßARA5	nßGAA5	ßMAN5	AST
J	dA	P	C	R	GG	AA	DA	AcKR	VTS	ALYL	LANA	ENAOH	ßARA7	tßGAL7	ßdFUC7	aMAN7	aAMY7	CATS7	PNPG4	aMAN4	PNPX5	aGLU5	PGUR5	HIP
K	IA	HP	Y	BzdIR	GA	dAdA	DK	HS	PKA	APAR	DANA	KNA	aARAF7	2vßGAL	ßtFUC7	ßMAN7	t5MP7	oACET7	ßdFUC4	PGUR4	PNPG5	ßGLU5	aRHA5	IND
L	ßA	F	Q	BzR	GP	AR	DR	GPL	FPA	AFPA	VNA	RNA	PNPX7	GAL6P7	aGLU7	PGUR7	DIP7	pACET7	nßGAA4	ßCEL4	aGAL5	aCHIT5	ßCEL5	NTA
M	V	GyF	N	H	GF	LG	GyDK	GyGGF	sFGL	AcAAPA	LNA	BzRNA	ONPX7	nßGAA7	ßGLU7	aRHA7	PHOS7	BUT7	aGLU4	PHOS4	oaGAL5	CHIT5	aMAL5	NTI
N	V4M	W	D	Orn	GdF	PR	EE	AAF	sYGL	LLVYS	ENA	PYRNA	aXYL7	naGAA7	tßGLU7	ßCEL7	BISPH7	PHOS8	ßGLU4	aARA5	ßdFUC5	aMAN5	PHOS5	PCA
O	IL	M	E	Pyr	GW	FP	EH	APA	HLH	G4DK	PNA	GPNA	PNPG7	onGAA7	aCHIT7	aMAL7	PHCHO7	TTR	ESC	DECO	ADH	LDC	UREI	PCC
P	dL	S	EßNa	Hyp	GR	FR	RR	AFP	PyHP	PepK	FNA	AFPNA	aGAL7	aIFUC7	CHIT7	ßMAL7	SULF7	VP	H2S	H2S/ESCO	GDC	ODC	UREII	TDA

Ac-Gly-Lys-ß	AcGK	D-Ala-D-Ala-ß	dAdA	His-ßNA	H	Trp-ß	W	p-nitrophenyl-	aXYL7	p-nitrophenyl-ß-	ßtFUC7
Glu-Glu-ß	EE	D-Ala-ß	dA	Hyp-ßNA-HCl	Hyp	Tryptophandeaminase	TDA	a-d-xylopyranosid		d-thiofucopyranosid	
2-meth.-4-(2-nitrovinyl)-	2vßGAL	D-Leu-ß	dL	Ile-ß	I	Tyr-ß	Y	p-nitrophenyl-	aARAF	p-nitrophenyl-	PNPX5
ph.-ß-d-gal.p.		DNA	DNA	Indol	IND	Urease I	UREI	a-l-arabinofuranosid		ß-d-xylopyranosid	
2´deoxythymidin-	DIP7	Decarboxylase Kontr.	DECO	L-Ala-pNA	LANA	Urease II	UREII	p-nitrophenyl-	aARA5	p-nitrophenyl-	PNPX4
5´-p-NP-phosphat		Esculin	ESC	Leu-Gly-ß	LG	Val-Tyr-Ser-ß	VTS	a-l-arabinosid		ß-dxylopyranosid	
3-indoxylsulfat	INDS7	Gln-ß	Q	Leu-pNA	LNA	Val-pNA	VNA	p-nitrophenyl-	alFUC7	p-nitrophenyl-	ßARA5
4-nitro-a-d-maltoheptaosia	AMY7	Glu(pNA)-OH	ENAOH	Leu-ß	IL	Val-ß	V	a-l-fucopyranosid		ß-l-arabinopyranosid	
4,6,o-ethyliden		Glu(ßNA)-OH	EßNa	LeuLeuValTyrSer-ß	LLVYS	Voges-Proskauer	VP	p-nitrophenyl-	aRHA5	p-nitrophenyl-	ßlFUC7
Ac-Ala-Ala-Pro-Ala-ß	AcAAPA	Glu-His-ß	EH	Lys-pNA	KNA	Medium		a-l-rhamnopyranosid		ß-l-fucopyranosid	
Ac-Lys-Ala-ß	AcKA	Glu-pNA	ENA	Lys-ß	K	bis-p-nitrophenylph.	BISPH7	p-nitrophenyl-n-acetyl-1-t	nGLUA	p-nitrophenylphophat	PHOS5
Ac-Lys-Arg-ß	AcKR	Glu-ß	E	Lysindecarboxylase	LDC	d-Ala-pNA	DANA	ß-d-glucosaminid		di(2-amoni-2-ethyl-	
Ala-Ala-Ala-pNA	AAA	Gluta.-Gly-Gly-Phe-ß	GyGGF	Nitrat Reduktion	NTA	o-nitrophenyl acetat	oACET	p-nitrophenyl-n-acetyl-	aCHIT4	p-nitrophenylphospha	PHOS4
Ala-Ala-Phe-ß	AAF	Glutamins. Decarbox.	GDC	Nitrit Reduktion	NTI	o-nitrophenyl-	oaGAL	a-d-glucosaminid		t di(2-amino-2-2ethy	
Ala-Ala-ß	AA	Glutaryl-Asp-Lys-ß	GyDK	Orn-ß	Orn	a-d-galactopyranosid		p-nitrophenyl-n-acetyl-	nßGAA	p-nitrophenylphospha	PHOS7
Ala-Arg-ß	AR	Gly-Ala-ß	GA	Ornithindecarboxyl.	ODC	o-nitrophenyl-n-acetyl-	naGAA	ß-d-galactosamid		t di(2-amino-2-ethyl	
Ala-Leu-Tyr-Leu-ß	ALYL	Gly-Arg-ß	GR	Peptidase Kontrolle	PepK	a-d-galactosaminid		p-nitrophenyl-n-acetyl-	nßGAA	p-nitrophenylphosphat	PHOS8
Ala-Phe-Pro-Ala-ß	AFPA	Gly-Asp-	G4DK	Phe-Arg-ß	FR	o-nitrophenyl-n-acetyl-	onGAA	ß-d-galactosaminid		pH=8,2	
Ala-Phe-Pro-pNA	AFPNA	Asp-Asp-Asp-Lys-ß		Phe-Pro-Ala-ß	FPA	ß-d-galactosaminid		p-nitrophenyl-n-acetyl-	CHIT5	p-nitrophenylsulfat	SULF7
Ala-Phe-Pro-ß	AFP	Gly-Gly-ß	GG	Phe-Pro-ß	FP	o-nitrophenyl-ß-d-	GAL6P	ß-d-glucosaminid		thymidin-5´-monophosp	t5MP7
Ala-Pro-Ala-Arg-ß	APAR	Gly-Phe-ß	GF	Phe-pNA	FNA	gal.pyr.-6-phosphat		p-nitrophenyl-	PHCHO	p-NP	
Ala-Pro-Ala-ß	APA	Gly-Pro-Leu-ß	GPL	Phe-ß	F	o-nitrophenyl-	ONPX7	phosphorycholin		ß-Ala-ßNA	ßA
Ala-ß	lA	Gly-Pro-pNA	GPNA	Pro-Arg-ß	PR	ß-d-xylopyranosid		p-nitrophenyl-	ßCEL4		
Arg-Arg-ß	RR	Gly-Pro-ß	GP	Pro-Lys-Ala	PKA	p-nitrocatecholsulf.	CATS7	ß-d-cellobiosid			
Arg-pNA	RNA	Gly-Trp-ß	GW	Pro-pNA	PNA	p-nitrophenyl acetat	pACET	p-nitrophenyl-	ßdFUC		
Arg-ß	R	Gly-d-Phe-ß	GdF	Pro-ß	P	p-nitrophenyl butyr.	BUT7	ß-d-fucopyranosid			
Arginindihydrolase	ADH	Gly-pNA	GNA	Pyr-His(Bzl)-Pro-ß	PyHBP	p-nitrophenyl-1-thio-	tßGAL7	p-nitrophenyl-	PNPG4		
Arylsulfatase	AST	Gly-ß	G	Pyr-His-Pro-ß	PyHP	ß-d-galactopyranosid		ß-d-galactopyranosid			
Asn-ß	N	Glytaryl-Phe-ß	GyF	Pyr-pNA	PYRNA	p-nitrophenyl-1-thio-	tßGLU7	p-nitrophenyl-	ßGLU4		
Asp-Ala-ß	DA	H-DL-Met-ßNA	M	Pyr-ß	Pyr	ß-d-glucopyranosid		ß-d-glucopyranosid			
Asp-Arg-ß	DR	H-Hydroxyprolin-ßNA	HP	Pyrazinamidase	PCA	p-nitrophenyl-	aGAL5	p-nitrophenyl-	PGUR4		
Asp-Lys-ß	DK	H-Val-4-MßNA	V4M	Pyrazinamidase Kont.	PCC	a-d-galactopyranosid		ß-d-glucuronid			
Asp-ß	D	H2S Medium	H2S	Ser-Tyr-ß	SY	p-nitrophenyl-	aGLU4	p-nitrophenyl-	ßLAC7		
Bz-Arg-ß	BzR	H2S/	H2S/	Ser-ß	S	a-d-glucopyranosid		ß-d-lactopyranosid			
Bz-DL-Arg-pNA	BzRNA	Esculin Kontrolle	ESCO	Suc-Phe-Gly-Leu-ß	sFGL	p-nitrophenyl-	aMAL5	p-nitrophenyl-	ßMAL7		
Bz-DL-Arg-ß	BzdlR	HIP	HIP	Suc-Tyr-Gly-Leu-ß	sYGL	a-d-maltosid		ß-d-maltosid			
Citrat	CIT	His-Leu-His-ßNA	HLH	Tetrathionatreduct.	T	p-nitrophenyl-	aMAN4	p-nitrophenyl-	ßMAN5		
Cys-ß	C	His-Ser-ßNA	HS	Thr-ß	T	a-d-mannopyranosid		ß-d-mannopyranosid			
