# Supplementary figures and images for: Differential phenotyping of Brucella species using a newly developed semi-automated metabolic system
Source: BMC Microbiol. 2010 Oct 23;10:269. doi: 10.1186/1471-2180-10-269 (PMC2984481; doi:10.1186/1471-2180-10-269)

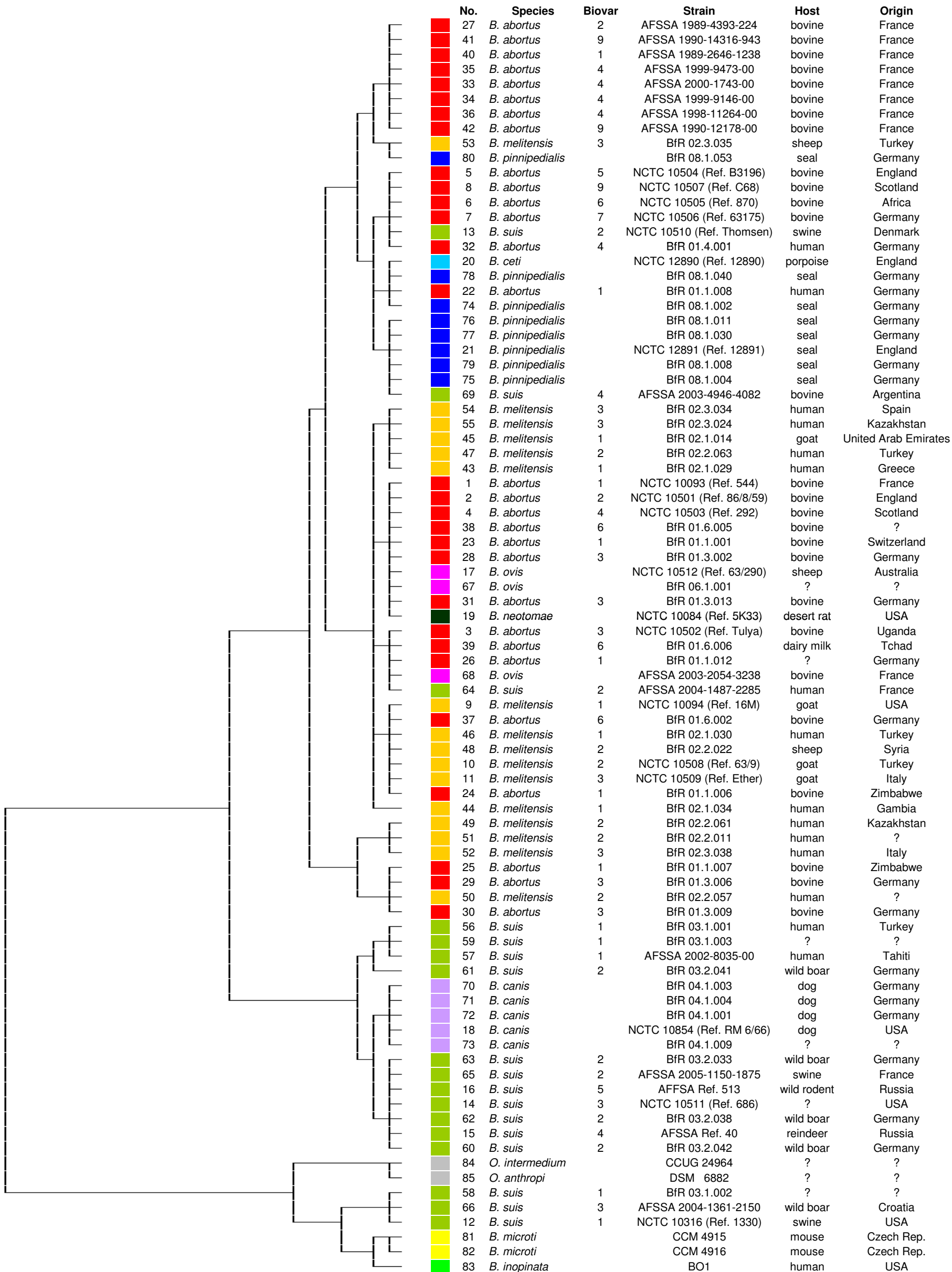

Supplement: Additional file 4 — Cluster analysis of Brucella reference and field strains based on their amino acid metabolism. Cluster analysis of 83 Brucella and 2 Ochrobactrum strains based on 191 biochemical reactions tested with the Taxa Profile™ A plate. Hierarchical cluster analysis was performed by the Ward's linkage algorithm using the raw OD data. [file 1471-2180-10-269-S4.PDF]

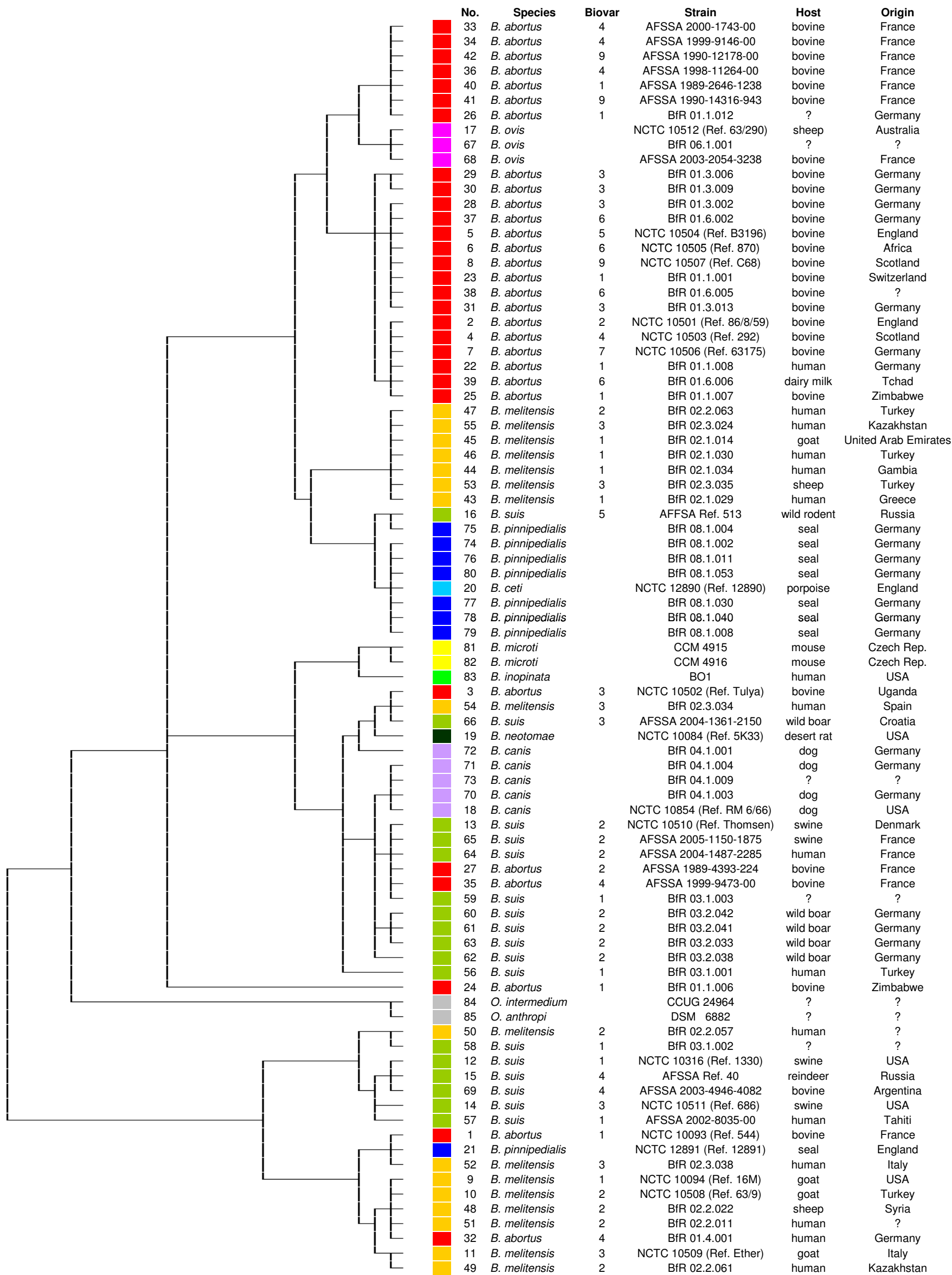

Supplement: Additional file 5 — Cluster analysis of Brucella reference and field strains based on their carbohydrate metabolism. Cluster analysis of 83 Brucella and 2 Ochrobactrum strains based on 191 biochemical reactions tested with the Taxa Profile™ C plate. Hierarchical cluster analysis was performed by the Ward's linkage algorithm using the raw OD data. [file 1471-2180-10-269-S5.PDF]

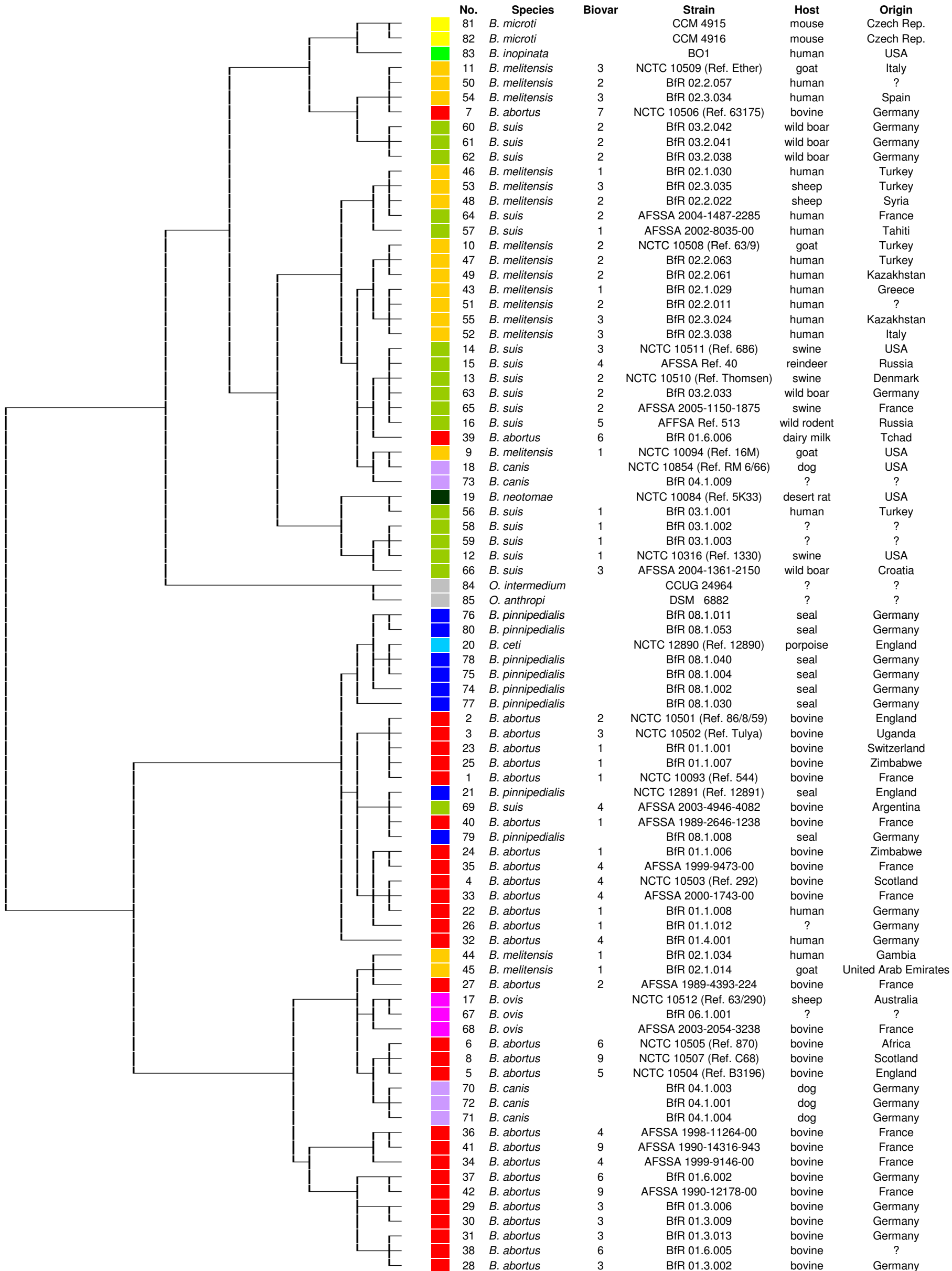

Supplement: Additional file 6 — Cluster analysis of Brucella reference and field strains based on specific enzymatic reactions. Cluster analysis of 83 Brucella and 2 Ochrobactrum strains based on 188 biochemical reactions tested with the Taxa Profile™ E plate. Hierarchical cluster analysis was performed by the Ward's linkage algorithm using the raw OD data. [file 1471-2180-10-269-S6.PDF]
