# Supplementary material for: Differential phenotyping of Brucella species using a newly developed semi-automated metabolic system
Source: BMC Microbiol. 2010 Oct 23;10:269. doi: 10.1186/1471-2180-10-269 (PMC2984481; doi:10.1186/1471-2180-10-269)
Supplement: Additional file 7 — Metabolic activity of Brucella strains. Relative frequency (%) of positive and negative metabolic activity among 23 Brucella reference strains and 90 field isolates (Table 2) observed for the 93 substances tested in the Brucella specific Micronaut™ plate. Both quality and relative quantity are presented: - no metabolic activity (highlighted in green), + moderate metabolic activity (in orange), ++ strong metabolic activity (in red). [file 1471-2180-10-269-S7.PDF]

	B. abortus bv 1	*B. abortus* bv 2	*B. abortus* bv 3	*B. abortus* bv 4	*B. abortus* bv 5	*B. abortus* bv 6	*B. abortus* bv 7	*B. abortus* bv 9	*B. melitensis* bv 1	*B. melitensis* bv 2	*B. melitensis* bv 3	*B. suis* bv 1	*B. suis* bv 2	*B. suis* bv 3	*B. suis* bv 4	*B. suis* bv 5	*B. ovis*	*B. canis*	*B. neotomae*	*B. ceti*	*B. pinnipedialis*	*B. microti*	*B. inopinata*	*Ochrobactrum* spp.	*Acinetobacter lwoffii*	*Y. enterocolitica* O:9	*Brucella* unknown
DANA	++ (100%)	++ (100%)	++ (100%)	++ (100%)	++ (100%)	++ (100%)	++ (100%)	++ (100%)	++ (100%)	++ (100%)	++ (100%)	++ (100%)	++ (100%)	++ (100%)	++ (100%)	+ (100%)	++ (100%)	++ (100%)	- (100%)	++ (100%)	++ (100%)	++ (100%)	++ (100%)	++ (100%)	- (100%)	- (100%)	- (100%)
GNA	++ (100%)	++ (100%)	++ (100%)	++ (100%)	++ (100%)	++ (100%)	++ (100%)	++ (100%)	++ (100%)	++ (100%)	++ (100%)	++ (100%)	++ (100%)	++ (100%)	++ (100%)	+ (100%)	++ (100%)	++ (100%)	- (100%)	++ (100%)	++ (100%)	++ (100%)	++ (100%)	++ (100%)	- (100%)	- (100%)	- (100%)
LNA	+ (100%)	+ (100%)	+ (100%)	+ (83,3%)	+ (100%)	+ (100%)	+ (100%)	+ (100%)	+ (100%)	+ (100%)	+ (100%)	+ (100%)	+ (100%)	+ (100%)	+ (100%)	+ (100%)	+ (100%)	+ (100%)	- (100%)	+ (100%)	+ (93,8%)	+ (100%)	++ (100%)	+ (100%)	++ (100%)	+ (100%)	+ (100%)
KNA	+ (83,3%)	+ (100%)	++ (50%)	+ (100%)	+ (100%)	+ (60%)	++ (100%)	+ (100%)	++ (57,1%)	++ (100%)	++ (100%)	+ (66,7%)	++ (100%)	+ (100%)	++ (100%)	++ (100%)	+ (100%)	++ (100%)	- (100%)	++ (100%)	++ (87,5%)	++ (100%)	++ (100%)	++ (100%)	++ (100%)	++ (100%)	++ (100%)
GPNA	- (100%)	- (100%)	- (100%)	- (100%)	- (100%)	- (100%)	- (100%)	- (100%)	++ (71,4%)	++ (50%)	++ (81,8%)	++ (83,3%)	++ (50%)	++ (100%)	++ (100%)	+ (100%)	- (100%)	++ (100%)	- (100%)	- (100%)	- (93,8%)	+ (100%)	++ (100%)	+ (50%)	- (100%)	- (100%)	- (100%)
AFPNA	- (100%)	- (100%)	- (100%)	- (100%)	- (100%)	- (100%)	- (100%)	- (100%)	+ (85,7%)	+ (100%)	+ (90,9%)	+ (83,3%)	+ (87,5%)	+ (100%)	+ (100%)	+ (100%)	- (100%)	+ (50%)	- (100%)	- (100%)	- (100%)	- (90%)	+ (100%)	- (100%)	- (100%)	- (100%)	- (100%)
ENAOH	- (100%)	- (100%)	- (100%)	- (100%)	- (100%)	- (100%)	- (100%)	- (100%)	- (100%)	- (100%)	- (100%)	- (83,3%)	- (100%)	- (100%)	- (100%)	- (100%)	- (100%)	- (100%)	- (100%)	- (100%)	- (100%)	- (100%)	- (100%)	++ (100%)	- (100%)	- (100%)	- (100%)
PYRNA	- (100%)	- (100%)	- (100%)	- (100%)	- (100%)	- (100%)	- (100%)	- (100%)	- (100%)	- (100%)	- (100%)	- (100%)	- (100%)	- (100%)	- (100%)	- (100%)	- (100%)	- (100%)	- (100%)	- (100%)	- (100%)	- (100%)	- (100%)	++ (100%)	- (100%)	- (100%)	- (100%)
HP	++ (100%)	++ (100%)	++ (100%)	++ (83,3%)	++ (100%)	++ (100%)	++ (100%)	++ (100%)	++ (100%)	++ (100%)	++ (100%)	++ (100%)	++ (100%)	++ (100%)	++ (100%)	++ (100%)	++ (100%)	++ (100%)	++ (100%)	++ (100%)	++ (100%)	++ (100%)	++ (100%)	++ (100%)	- (100%)	- (100%)	++ (100%)
IL	- (100%)	- (100%)	- (100%)	- (100%)	- (100%)	- (100%)	- (100%)	- (100%)	- (100%)	- (100%)	- (100%)	- (100%)	- (100%)	- (100%)	- (100%)	- (100%)	- (100%)	- (100%)	- (100%)	- (100%)	- (100%)	- (100%)	- (100%)	+ (50%)	- (100%)	- (100%)	- (100%)
K	++ (66,7%)	++ (100%)	++ (100%)	++ (100%)	++ (100%)	++ (100%)	++ (100%)	++ (100%)	++ (100%)	++ (100%)	++ (100%)	+ (100%)	++ (100%)	++ (100%)	++ (100%)	++ (100%)	++ (100%)	++ (75%)	- (100%)	++ (100%)	++ (87,5%)	++ (100%)	++ (100%)	++ (100%)	++ (100%)	++ (100%)	++ (100%)
RR	+ (100%)	+ (100%)	++ (100%)	+ (100%)	+ (100%)	+ (60%)	++ (100%)	++ (66,7%)	+ (100%)	++ (64,3%)	++ (54,5%)	+ (66,7%)	++ (100%)	++ (100%)	++ (100%)	++ (100%)	++ (100%)	++ (75%)	+ (100%)	+ (100%)	- (62,5%)	++ (50%)	++ (100%)	++ (100%)	++ (100%)	++ (100%)	+ (100%)
GG	++ (100%)	++ (100%)	++ (100%)	++ (100%)	++ (100%)	++ (100%)	++ (100%)	++ (100%)	++ (100%)	++ (100%)	++ (100%)	++ (100%)	++ (100%)	++ (100%)	++ (100%)	++ (100%)	++ (100%)	++ (100%)	- (100%)	++ (100%)	++ (100%)	++ (100%)	++ (100%)	++ (100%)	++ (100%)	++ (100%)	++ (100%)
P	++ (100%)	++ (100%)	++ (100%)	++ (100%)	++ (100%)	++ (100%)	++ (100%)	++ (100%)	++ (100%)	++ (100%)	++ (100%)	++ (100%)	++ (100%)	++ (100%)	++ (100%)	++ (100%)	++ (100%)	++ (100%)	++ (100%)	++ (100%)	++ (100%)	++ (100%)	++ (100%)	++ (100%)	- (100%)	- (100%)	++ (100%)
AcGK	- (100%)	- (100%)	- (100%)	- (100%)	- (100%)	- (100%)	- (100%)	- (100%)	- (71,4%)	+ (57,1%)	+ (72,7%)	+ (83,3%)	+ (87,5%)	+ (100%)	+ (100%)	+ (100%)	++ (100%)	++ (75%)	+ (100%)	- (100%)	- (100%)	- (90%)	++ (100%)	+ (100%)	+ (100%)	- (100%)	+ (50%)
AFPA	+ (100%)	++ (100%)	+ (100%)	+ (83,3%)	+ (100%)	+ (60%)	+ (100%)	+ (100%)	++ (100%)	++ (100%)	++ (100%)	++ (100%)	++ (100%)	++ (100%)	++ (100%)	++ (100%)	+ (50%)	++ (100%)	++ (100%)	++ (100%)	++ (56,3%)	+ (80%)	++ (100%)	++ (50%)	++ (100%)	++ (100%)	++ (100%)
N	+ (100%)	+ (50%)	+ (100%)	+ (100%)	+ (100%)	+ (80%)	+ (100%)	+ (100%)	+ (85,7%)	+ (100%)	+ (90,9%)	++ (66,7%)	++ (62,5%)	++ (100%)	++ (100%)	+ (100%)	+ (75%)	+ (75%)	+ (100%)	+ (100%)	+ (62,5%)	- (100%)	++ (100%)	- (100%)	+ (100%)	- (100%)	++ (100%)
W	++ (100%)	++ (100%)	++ (100%)	++ (100%)	- (100%)	++ (100%)	++ (100%)	+ (66,7%)	++ (71,4%)	++ (100%)	++ (100%)	++ (100%)	++ (100%)	- (100%)	- (100%)	++ (100%)	- (100%)	- (100%)	++ (100%)	++ (100%)	++ (100%)	++ (90%)	++ (100%)	++ (50%)	+ (100%)	- (100%)	- (100%)
EH	+ (100%)	+ (100%)	+ (100%)	+ (83,3%)	+ (100%)	+ (60%)	+ (100%)	+ (100%)	+ (100%)	+ (100%)	+ (72,7%)	+ (66,7%)	++ (100%)	+ (100%)	+ (100%)	+ (100%)	- (100%)	+ (75%)	+ (100%)	++ (100%)	- (81,3%)	+ (90%)	++ (100%)	+ (100%)	- (100%)	- (100%)	+ (100%)
AcKA	- (100%)	- (100%)	- (100%)	- (100%)	- (100%)	- (100%)	- (100%)	- (100%)	- (71,4%)	+ (71,4%)	+ (63,6%)	+ (100%)	+ (100%)	+ (100%)	+ (100%)	+ (100%)	++ (75%)	++ (50%)	+ (100%)	- (100%)	- (100%)	- (100%)	++ (100%)	+ (100%)	- (100%)	- (100%)	- (100%)
dAdA	+ (100%)	+ (100%)	+ (100%)	+ (100%)	+ (100%)	+ (100%)	+ (100%)	+ (100%)	- (71,4%)	- (71,4%)	+ (72,7%)	+ (83,3%)	+ (100%)	+ (100%)	+ (100%)	- (100%)	- (100%)	+ (50%)	- (100%)	+ (100%)	+ (81,3%)	+ (100%)	++ (100%)	++ (100%)	- (100%)	- (100%)	- (100%)
V	- (100%)	- (100%)	- (100%)	- (100%)	- (100%)	- (100%)	- (100%)	- (100%)	- (100%)	- (100%)	- (100%)	- (100%)	+ (87,5%)	- (100%)	- (100%)	- (100%)	- (100%)	- (100%)	- (100%)	- (100%)	- (93,8%)	- (100%)	- (100%)	- (100%)	+ (100%)	- (100%)	- (100%)
I	- (100%)	- (100%)	- (100%)	- (100%)	- (100%)	- (100%)	- (100%)	- (100%)	- (100%)	- (100%)	- (100%)	- (100%)	- (100%)	- (100%)	- (100%)	- (100%)	- (100%)	- (100%)	- (100%)	- (100%)	- (100%)	- (100%)	- (100%)	- (100%)	+ (100%)	- (100%)	- (100%)
VTS	- (100%)	+ (50%)	- (100%)	- (100%)	- (100%)	- (100%)	- (100%)	- (100%)	- (71,4%)	+ (71,4%)	+ (81,8%)	+ (100%)	++ (75%)	+ (100%)	+ (100%)	++ (100%)	+ (100%)	+ (50%)	- (100%)	- (100%)	- (56,3%)	+ (50%)	++ (100%)	+ (100%)	+ (100%)	- (100%)	+ (100%)
D	- (100%)	- (100%)	- (100%)	- (100%)	- (100%)	- (100%)	- (100%)	- (100%)	- (100%)	- (100%)	- (100%)	- (100%)	- (100%)	- (100%)	- (100%)	- (100%)	- (100%)	- (100%)	- (100%)	- (100%)	- (100%)	- (100%)	- (100%)	- (100%)	++ (100%)	- (100%)	- (100%)
E	- (100%)	- (100%)	- (100%)	- (100%)	- (100%)	- (100%)	- (100%)	- (100%)	- (100%)	- (100%)	- (100%)	- (100%)	- (100%)	- (100%)	- (100%)	- (100%)	- (100%)	- (100%)	- (100%)	- (100%)	- (100%)	- (100%)	- (100%)	- (100%)	++ (100%)	+ (100%)	- (100%)
Pyr	- (100%)	- (100%)	- (100%)	- (100%)	- (100%)	- (100%)	- (100%)	- (100%)	- (100%)	- (100%)	- (100%)	- (100%)	- (100%)	- (100%)	- (100%)	- (100%)	- (100%)	- (100%)	- (100%)	- (100%)	- (100%)	- (100%)	- (100%)	++ (100%)	- (100%)	++ (100%)	- (100%)
βA	- (100%)	- (100%)	- (100%)	- (100%)	- (100%)	- (100%)	- (100%)	- (100%)	- (100%)	- (100%)	- (100%)	- (100%)	- (100%)	- (100%)	- (100%)	- (100%)	- (100%)	- (100%)	- (100%)	- (100%)	- (100%)	- (100%)	- (100%)	+ (100%)	- (100%)	- (100%)	- (100%)
V4M	- (100%)	- (100%)	- (100%)	- (100%)	- (100%)	- (100%)	- (100%)	- (100%)	- (100%)	- (100%)	- (100%)	- (100%)	- (100%)	- (100%)	- (100%)	- (100%)	- (100%)	- (100%)	- (100%)	- (100%)	- (100%)	- (100%)	- (100%)	- (100%)	- (100%)	- (100%)	- (100%)
PepK	- (100%)	- (100%)	- (100%)	- (100%)	- (100%)	- (100%)	- (100%)	- (100%)	- (100%)	- (100%)	- (100%)	- (100%)	- (100%)	- (100%)	- (100%)	- (100%)	- (100%)	- (100%)	- (100%)	- (100%)	- (100%)	- (100%)	- (100%)	- (100%)	- (100%)	- (100%)	- (100%)
BISPH7	- (66,7%)	- (100%)	- (100%)	- (83,3%)	- (100%)	- (80%)	+ (100%)	- (100%)	- (71,4%)	- (64,3%)	- (63,6%)	++ (66,7%)	++ (87,5%)	+ (100%)	++ (100%)	+ (100%)	+ (50%)	+ (100%)	- (100%)	+ (100%)	++ (56,3%)	++ (100%)	++ (100%)	++ (100%)	- (100%)	++ (100%)	+ (100%)
PHOS7	- (66,7%)	- (100%)	- (100%)	- (100%)	- (100%)	+ (60%)	- (100%)	- (100%)	+ (100%)	+ (100%)	+ (90,9%)	++ (66,7%)	++ (62,5%)	+ (100%)	+ (100%)	+ (100%)	- (75%)	+ (75%)	- (100%)	+ (100%)	+ (100%)	++ (100%)	++ (100%)	+ (100%)	+ (100%)	++ (100%)	+ (100%)
aGLU7	- (100%)	- (100%)	- (100%)	- (100%)	- (100%)	- (100%)	- (100%)	- (100%)	- (71,4%)	+ (92,9%)	- (54,5%)	+ (66,7%)	++ (62,5%)	++ (100%)	++ (100%)	- (100%)	- (100%)	+ (100%)	+ (100%)	- (100%)	+ (50%)	++ (100%)	++ (100%)	++ (100%)	- (100%)	- (100%)	- (100%)
aMAL7	- (100%)	- (100%)	- (100%)	- (100%)	- (100%)	- (100%)	- (100%)	- (100%)	- (100%)	- (100%)	- (100%)	+ (50%)	- (100%)	- (100%)	- (100%)	- (100%)	- (100%)	- (100%)	- (100%)	- (100%)	- (100%)	+ (100%)	+ (100%)	+ (100%)	- (100%)	- (100%)	- (100%)
aGLU5	- (100%)	- (100%)	- (100%)	- (100%)	- (100%)	- (100%)	- (100%)	- (100%)	- (100%)	- (100%)	- (100%)	++ (50%)	+ (100%)	+ (100%)	+ (100%)	+ (100%)	- (100%)	+ (100%)	- (100%)	- (100%)	- (56,3%)	++ (100%)	++ (100%)	+ (100%)	- (100%)	++ (100%)	- (100%)
aXYL7	- (100%)	- (100%)	- (100%)	- (100%)	- (100%)	- (100%)	- (100%)	- (100%)	- (100%)	- (100%)	- (100%)	+ (100%)	+ (87,5%)	+ (100%)	+ (100%)	- (100%)	- (100%)	+ (50%)	- (100%)	- (100%)	- (87,5%)	+ (100%)	++ (100%)	+ (100%)	- (100%)	- (100%)	- (100%)
CHIT7	- (100%)	- (100%)	- (100%)	- (100%)	- (100%)	- (80%)	- (100%)	- (100%)	+ (100%)	+ (100%)	+ (100%)	+ (100%)	+ (100%)	++ (100%)	++ (100%)	+ (100%)	- (100%)	++ (50%)	+ (100%)	- (100%)	+ (100%)	++ (100%)	++ (100%)	++ (100%)	- (100%)	++ (100%)	++ (100%)
EROL	++ (100%)	++ (100%)	++ (100%)	++ (100%)	++ (100%)	++ (100%)	++ (100%)	++ (100%)	++ (100%)	++ (100%)	++ (100%)	+ (66,7%)	++ (100%)	++ (100%)	++ (100%)	++ (100%)	- (100%)	+ (100%)	++ (100%)	++ (100%)	++ (50%)	++ (100%)	++ (100%)	++ (100%)	- (100%)	- (100%)	++ (100%)
L-FUC	- (100%)	- (100%)	- (100%)	+ (66,7%)	++ (100%)	+ (80%)	++ (100%)	- (100%)	++ (100%)	++ (100%)	++ (100%)	++ (100%)	- (100%)	- (100%)	++ (100%)	++ (100%)	- (100%)	++ (50%)	- (100%)	++ (100%)	- (75%)	++ (100%)	++ (100%)	++ (100%)	- (100%)	- (100%)	+ (100%)
GLUCY	++ (100%)	++ (100%)	++ (100%)	++ (100%)	++ (100%)	++ (100%)	++ (100%)	++ (100%)	++ (100%)	++ (100%)	++ (100%)	++ (100%)	++ (100%)	++ (100%)	++ (100%)	++ (100%)	+ (100%)	++ (100%)	++ (100%)	++ (100%)	++ (100%)	++ (100%)	++ (100%)	++ (100%)	- (100%)	++ (100%)	++ (100%)
D-RIB	++ (100%)	++ (100%)	++ (100%)	++ (100%)	++ (100%)	++ (100%)	++ (100%)	++ (100%)	- (71,4%)	- (100%)	- (100%)	++ (100%)	++ (100%)	++ (100%)	++ (100%)	++ (100%)	- (100%)	++ (50%)	- (100%)	++ (100%)	- (100%)	++ (100%)	++ (100%)	++ (100%)	- (100%)	++ (100%)	+ (100%)
D-ARA	- (100%)	- (100%)	- (100%)	- (100%)	- (100%)	- (60%)	++ (100%)	- (100%)	+ (85,7%)	++ (71,4%)	+ (72,7%)	+ (66,7%)	- (100%)	- (100%)	- (100%)	- (100%)	- (100%)	+ (100%)	- (100%)	- (100%)	- (100%)	+ (60%)	++ (100%)	++ (100%)	- (100%)	- (100%)	- (100%)
D-GLU	++ (100%)	++ (100%)	++ (100%)	++ (100%)	++ (100%)	++ (100%)	++ (100%)	++ (100%)	++ (100%)	++ (100%)	++ (100%)	++ (100%)	++ (100%)	++ (100%)	++ (100%)	++ (100%)	- (100%)	++ (100%)	++ (100%)	++ (100%)	- (62,5%)	++ (100%)	++ (100%)	++ (100%)	- (100%)	++ (100%)	++ (100%)
L-ARA	++ (100%)	++ (100%)	++ (100%)	++ (100%)	- (100%)	++ (100%)	++ (100%)	+ (66,7%)	++ (100%)	++ (100%)	++ (81,8%)	++ (100%)	++ (100%)	- (100%)	- (100%)	- (100%)	+ (50%)	+ (100%)	++ (100%)	++ (100%)	- (87,5%)	++ (100%)	++ (100%)	++ (100%)	- (100%)	++ (100%)	- (100%)
DOGAL	- (100%)	- (100%)	- (100%)	- (100%)	- (100%)	- (100%)	- (100%)	- (100%)	- (100%)	- (85,7%)	- (81,8%)	- (100%)	- (100%)	- (100%)	- (100%)	- (100%)	- (100%)	- (100%)	- (100%)	- (100%)	- (100%)	- (100%)	- (100%)	- (100%)	- (100%)	- (100%)	- (100%)
D-GAL	++ (100%)	++ (100%)	++ (100%)	++ (100%)	++ (100%)	++ (100%)	++ (100%)	++ (100%)	++ (100%)	++ (100%)	++ (81,8%)	++ (100%)	++ (100%)	- (100%)	- (100%)	- (100%)	- (100%)	+ (50%)	++ (100%)	++ (100%)	- (87,5%)	++ (100%)	++ (100%)	++ (100%)	- (100%)	++ (100%)	+ (100%)
D-XYL	++ (100%)	+ (100%)	+ (50%)	++ (100%)	- (100%)	+ (80%)	++ (100%)	+ (66,7%)	++ (100%)	++ (100%)	++ (100%)	++ (100%)	+ (75%)	++ (100%)	++ (100%)	++ (100%)	- (100%)	++ (50%)	++ (100%)	++ (100%)	- (100%)	++ (100%)	++ (100%)	++ (100%)	- (100%)	++ (100%)	++ (100%)
D-TAL	+ (100%)	+ (100%)	+ (50%)	++ (100%)	- (100%)	++ (100%)	++ (100%)	+ (66,7%)	+ (57,1%)	+ (85,7%)	- (63,6%)	+ (66,7%)	++ (100%)	- (100%)	- (100%)	- (100%)	- (100%)	+ (50%)	++ (100%)	++ (100%)	- (87,5%)	++ (100%)	- (100%)	++ (100%)	- (100%)	- (100%)	- (100%)
D-TOL	++ (66,7%)	++ (100%)	++ (50%)	++ (100%)	++ (100%)	++ (60%)	++ (100%)	++ (100%)	- (100%)	- (100%)	- (100%)	+ (66,7%)	++ (75%)	++ (100%)	- (100%)	++ (100%)	++ (50%)	++ (100%)	- (100%)	++ (100%)	+ (75%)	++ (100%)	++ (100%)	++ (100%)	- (100%)	- (100%)	- (100%)
ADON	++ (100%)	++ (100%)	++ (50%)	++ (100%)	++ (100%)	++ (100%)	++ (100%)	++ (100%)	++ (100%)	++ (100%)	++ (63,6%)	++ (100%)	++ (100%)	- (100%)	- (100%)	- (100%)	+ (100%)	- (100%)	++ (100%)	++ (100%)	++ (75%)	++ (100%)	- (100%)	++ (100%)	- (100%)	- (100%)	- (100%)
SUCR	- (100%)	- (100%)	- (100%)	- (100%)	- (100%)	- (100%)	- (100%)	- (100%)	- (100%)	- (100%)	- (100%)	++ (100%)	++ (100%)	++ (100%)	++ (100%)	- (100%)	- (100%)	++ (100%)	- (100%)	- (100%)	+ (87,5%)	++ (100%)	++ (100%)	++ (100%)	- (100%)	++ (100%)	- (100%)
MALTO	- (100%)	- (100%)	- (100%)	- (100%)	- (100%)	- (100%)	- (100%)	- (100%)	- (100%)	- (100%)	- (100%)	++ (100%)	++ (100%)	++ (100%)	++ (100%)	- (100%)	- (100%)	++ (100%)	- (100%)	- (100%)	++ (62,5%)	++ (100%)	++ (100%)	++ (100%)	- (100%)	- (100%)	- (100%)
D-FRU	++ (100%)	++ (100%)	+ (50%)	++ (100%)	++ (100%)	+ (80%)	- (100%)	+ (66,7%)	++ (100%)	++ (100%)	++ (100%)	++ (100%)	++ (100%)	++ (100%)	++ (100%)	++ (100%)	- (100%)	++ (100%)	++ (100%)	++ (100%)	+ (62,5%)	++ (100%)	++ (100%)	++ (100%)	- (100%)	++ (100%)	++ (100%)
DNSOL	- (100%)	- (100%)	- (100%)	- (100%)	- (100%)	- (100%)	- (100%)	- (100%)	- (100%)	- (100%)	- (100%)	- (100%)	- (100%)	- (100%)	- (100%)	- (100%)	- (100%)	- (100%)	- (100%)	- (100%)	- (100%)	- (100%)	- (100%)	++ (100%)	- (100%)	- (100%)	- (100%)
LYXA	- (100%)	- (100%)	- (100%)	- (100%)	- (100%)	- (100%)	- (100%)	- (100%)	- (100%)	- (100%)	- (100%)	++ (100%)	- (100%)	++ (100%)	++ (100%)	++ (100%)	- (100%)	++ (100%)	++ (100%)	++ (100%)	- (100%)	++ (100%)	++ (100%)	++ (100%)	- (100%)	- (100%)	++ (100%)
PAL	- (100%)	- (100%)	- (100%)	- (100%)	- (100%)	- (100%)	- (100%)	- (100%)	- (100%)	- (100%)	- (100%)	++ (100%)	++ (100%)	++ (100%)	++ (100%)	- (100%)	- (100%)	++ (100%)	- (100%)	- (100%)	+ (50%)	++ (100%)	++ (100%)	++ (100%)	- (100%)	- (100%)	- (100%)
INOL	+ (66,7%)	- (100%)	+ (50%)	- (66,7%)	- (100%)	- (60%)	- (100%)	- (100%)	+ (57,1%)	++ (57,1%)	- (63,6%)	- (100%)	- (100%)	- (100%)	- (100%)	- (100%)	- (100%)	- (100%)	- (100%)	- (100%)	- (100%)	- (100%)	++ (100%)	++ (100%)	- (100%)	++ (100%)	- (100%)
INON	- (100%)	- (100%)	+ (50%)	- (100%)	- (100%)	+ (80%)	- (100%)	+ (66,7%)	- (100%)	- (100%)	- (100%)	- (66,7%)	- (100%)	++ (100%)	++ (100%)	++ (100%)	- (100%)	++ (50%)	- (100%)	++ (100%)	+ (87,5%)	++ (100%)	- (100%)	++ (100%)	- (100%)	++ (100%)	++ (100%)
Galli	++ (100%)	++ (100%)	++ (100%)	++ (100%)	++ (100%)	++ (100%)	++ (100%)	++ (100%)	++ (100%)	++ (100%)	++ (100%)	++ (100%)	++ (100%)	++ (100%)	++ (100%)	++ (100%)	++ (100%)	++ (100%)	++ (100%)	++ (100%)	++ (100%)	++ (100%)	++ (100%)	++ (100%)	++ (100%)	++ (100%)	++ (100%)
diLac	++ (100%)	++ (100%)	++ (100%)	++ (100%)	++ (100%)	++ (100%)	++ (100%)	++ (100%)	++ (100%)	++ (100%)	++ (100%)	++ (100%)	++ (100%)	++ (100%)	++ (100%)	++ (100%)	++ (50%)	++ (100%)	++ (100%)	++ (100%)	++ (100%)	++ (100%)	++ (100%)	++ (100%)	++ (100%)	- (100%)	++ (100%)
Acet	- (100%)	- (100%)	+ (50%)	- (100%)	- (100%)	- (100%)	- (100%)	+ (66,7%)	- (100%)	- (85,7%)	- (100%)	++ (66,7%)	++ (100%)	++ (100%)	++ (100%)	++ (100%)	++ (100%)	++ (100%)	++ (100%)	- (100%)	- (87,5%)	++ (100%)	++ (100%)	++ (100%)	++ (100%)	- (100%)	- (100%)
L-Asn	++ (100%)	++ (100%)	++ (50%)	++ (100%)	++ (100%)	++ (60%)	++ (100%)	++ (100%)	++ (100%)	++ (100%)	++ (100%)	+ (100%)	++ (100%)	- (100%)	++ (100%)	++ (100%)	- (100%)	++ (100%)	- (100%)	- (100%)	- (87,5%)	++ (100%)	++ (100%)	++ (100%)	- (100%)	++ (100%)	- (100%)
L-Glu	++ (100%)	++ (100%)	++ (50%)	++ (100%)	++ (100%)	++ (100%)	++ (100%)	+ (66,7%)	++ (100%)	++ (100%)	++ (100%)	++ (66,7%)	++ (50%)	++ (100%)	++ (100%)	++ (100%)	++ (50%)	++ (100%)	++ (100%)	++ (100%)	+ (87,5%)	++ (100%)	++ (100%)	++ (100%)	- (100%)	++ (100%)	++ (100%)
AlaGln	++ (100%)	++ (100%)	++ (50%)	++ (100%)	++ (100%)	++ (100%)	++ (100%)	+ (66,7%)	++ (71,4%)	++ (85,7%)	+ (54,5%)	++ (66,7%)	+ (50%)	++ (100%)	++ (100%)	- (100%)	- (100%)	+ (100%)	- (100%)	- (100%)	- (100%)	++ (100%)	++ (100%)	++ (100%)	- (100%)	++ (100%)	++ (100%)
GuaSu	- (100%)	+ (100%)	- (100%)	- (100%)	++ (100%)	- (60%)	- (100%)	+ (66,7%)	- (100%)	- (100%)	- (100%)	- (100%)	- (75%)	++ (100%)	- (100%)	++ (100%)	- (100%)	++ (100%)	- (100%)	++ (100%)	++ (100%)	++ (100%)	- (100%)	- (100%)	- (100%)	- (100%)	- (100%)
L-Cyss	++ (100%)	++ (100%)	++ (100%)	++ (100%)	++ (100%)	++ (100%)	++ (100%)	++ (100%)	++ (100%)	++ (100%)	++ (100%)	++ (100%)	++ (100%)	++ (100%)	++ (100%)	++ (100%)	++ (100%)	+ (50%)	++ (100%)	++ (100%)	++ (100%)	++ (100%)	++ (100%)	+ (100%)	- (100%)	- (100%)	++ (100%)
D-Ala	+ (66,7%)	- (100%)	+ (50%)	- (100%)	- (100%)	++ (60%)	++ (100%)	- (100%)	++ (71,4%)	++ (100%)	++ (100%)	+ (66,7%)	- (100%)	- (100%)	++ (100%)	- (100%)	- (100%)	- (100%)	++ (100%)	- (100%)	- (100%)	++ (60%)	++ (100%)	++ (100%)	++ (100%)	- (100%)	- (100%)
Propn	- (100%)	- (100%)	+ (50%)	- (100%)	- (100%)	- (60%)	- (100%)	- (100%)	++ (100%)	++ (100%)	++ (100%)	- (66,7%)	+ (75%)	- (100%)	- (100%)	++ (100%)	- (100%)	+ (100%)	++ (100%)	- (100%)	+ (75%)	- (100%)	++ (100%)	++ (100%)	++ (100%)	- (100%)	- (100%)
L-Ala	+ (100%)	- (100%)	+ (100%)	- (66,7%)	- (100%)	++ (60%)	++ (100%)	- (100%)	++ (100%)	++ (100%)	++ (100%)	+ (66,7%)	- (75%)	- (100%)	++ (100%)	- (100%)	- (100%)	++ (50%)	- (100%)	- (100%)	- (100%)	++ (100%)	++ (100%)	++ (100%)	++ (100%)	++ (100%)	- (100%)
βHBut	- (100%)	- (100%)	- (100%)	- (100%)	- (100%)	- (100%)	- (100%)	- (100%)	- (100%)	- (100%)	- (100%)	++ (66,7%)	- (100%)	++ (100%)	++ (100%)	++ (100%)	- (100%)	++ (100%)	++ (100%)	- (100%)	- (100%)	++ (60%)	++ (100%)	++ (100%)	++ (100%)	- (100%)	- (100%)
D-Asn	- (100%)	- (100%)	- (100%)	- (100%)	++ (100%)	- (60%)	++ (100%)	+ (66,7%)	- (71,4%)	+ (57,1%)	- (63,6%)	- (100%)	- (100%)	- (100%)	- (100%)	- (100%)	- (100%)	- (100%)	- (100%)	- (100%)	- (100%)	- (100%)	- (100%)	+ (100%)	- (100%)	- (100%)	- (100%)
L-Arg	- (100%)	- (100%)	+ (50%)	- (100%)	- (100%)	- (100%)	- (100%)	- (100%)	- (100%)	- (100%)	- (100%)	++ (100%)	++ (75%)	++ (100%)	++ (100%)	- (100%)	- (100%)	++ (100%)	- (100%)	- (100%)	- (100%)	++ (100%)	++ (100%)	++ (100%)	- (100%)	- (100%)	+ (100%)
AcArg	- (100%)	- (100%)	- (100%)	- (100%)	- (100%)	- (100%)	- (100%)	- (100%)	- (100%)	- (100%)	- (100%)	++ (100%)	++ (75%)	++ (100%)	++ (100%)	- (100%)	- (100%)	++ (100%)	- (100%)	- (100%)	- (100%)	+ (80%)	++ (100%)	++ (100%)	- (100%)	- (100%)	+ (100%)
Glyx	- (100%)	- (100%)	- (100%)	- (100%)	- (100%)	- (100%)	- (100%)	- (100%)	- (100%)	- (100%)	- (81,8%)	- (100%)	+ (75%)	- (100%)	- (100%)	- (100%)	- (100%)	+ (50%)	- (100%)	- (100%)	- (100%)	- (100%)	++ (100%)	+ (100%)	- (100%)	- (100%)	- (100%)
L-Ser	- (100%)	- (100%)	- (100%)	- (100%)	- (100%)	- (60%)	- (100%)	- (100%)	++ (71,4%)	++ (100%)	++ (81,8%)	+ (100%)	+ (100%)	- (100%)	++ (100%)	- (100%)	+ (100%)	+ (50%)	- (100%)	- (100%)	- (100%)	++ (100%)	++ (100%)	++ (100%)	- (100%)	++ (100%)	- (100%)
HipArg	- (100%)	- (100%)	- (100%)	- (100%)	- (100%)	- (100%)	- (100%)	- (100%)	- (100%)	- (100%)	- (100%)	++ (66,7%)	++ (75%)	++ (100%)	++ (100%)	- (100%)	- (100%)	+ (100%)	- (100%)	- (100%)	- (100%)	+ (80%)	++ (100%)	++ (100%)	- (100%)	- (100%)	- (100%)
L-Caro	+ (66,7%)	- (100%)	+ (50%)	- (100%)	- (100%)	+ (80%)	- (100%)	- (100%)	- (100%)	- (100%)	- (100%)	++ (100%)	- (100%)	++ (100%)	- (100%)	- (100%)	- (100%)	- (100%)	- (100%)	- (100%)	- (100%)	++ (100%)	++ (100%)	++ (100%)	- (100%)	- (100%)	- (100%)
Gly	- (100%)	- (100%)	- (100%)	- (100%)	- (100%)	- (100%)	- (100%)	- (100%)	- (100%)	- (100%)	- (100%)	- (66,7%)	- (100%)	- (100%)	- (100%)	- (100%)	- (100%)	- (100%)	- (100%)	- (100%)	- (100%)	++ (60%)	++ (100%)	+ (100%)	- (100%)	- (100%)	- (100%)
Adipa	- (100%)	- (100%)	- (100%)	- (100%)	- (100%)	- (100%)	- (100%)	- (100%)	- (100%)	- (100%)	- (100%)	++ (100%)	- (100%)	- (100%)	- (100%)	++ (100%)	- (100%)	+ (50%)	- (100%)	++ (100%)	- (100%)	++ (100%)	- (100%)	+ (100%)	++ (100%)	- (100%)	- (100%)
L-Pro	- (66,7%)	- (100%)	+ (50%)	- (100%)	- (100%)	- (60%)	- (100%)	- (100%)	++ (71,4%)	++ (100%)	++ (100%)	- (66,7%)	- (100%)	- (100%)	- (100%)	- (100%)	- (100%)	- (100%)	- (100%)	- (100%)	- (75%)	++ (100%)	++ (100%)	++ (100%)	++ (100%)	++ (100%)	- (100%)
D-Pro	- (100%)	- (100%)	- (100%)	- (100%)	- (100%)	- (60%)	- (100%)	- (100%)	+ (85,7%)	++ (100%)	++ (100%)	- (100%)	+ (75%)	- (100%)	- (100%)	- (100%)	- (100%)	+ (50%)	- (100%)	- (100%)	- (100%)	++ (100%)	++ (100%)	++ (100%)	- (100%)	- (100%)	- (100%)
Fuma	- (100%)	- (100%)	+ (50%)	- (66,7%)	- (100%)	- (60%)	- (100%)	- (100%)	- (100%)	- (85,7%)	- (100%)	- (100%)	- (75%)	- (100%)	- (100%)	- (100%)	- (100%)	- (100%)	- (100%)	- (100%)	- (100%)	++ (100%)	++ (100%)	+ (100%)	++ (100%)	++ (100%)	- (100%)
D-Ser	- (100%)	- (100%)	- (100%)	- (100%)	- (100%)	- (100%)	- (100%)	- (100%)	+ (57,1%)	++ (100%)	++ (81,8%)	- (100%)	- (100%)	- (100%)	- (100%)	- (100%)	- (100%)	- (100%)	- (100%)	- (100%)	- (100%)	- (100%)	- (100%)	- (100%)	- (100%)	- (100%)	- (100%)
Glyc	- (100%)	- (100%)	- (100%)	- (100%)	- (100%)	- (100%)	- (100%)	- (100%)	- (100%)	- (85,7%)	- (100%)	- (100%)	+ (100%)	- (100%)	- (100%)	- (100%)	- (100%)	++ (50%)	- (100%)	- (100%)	- (100%)	- (100%)	++ (100%)	++ (100%)	- (100%)	- (100%)	- (100%)
Adeni	++ (100%)	++ (100%)	++ (100%)	++ (100%)	++ (100%)	++ (100%)	++ (100%)	++ (100%)	++ (100%)	++ (100%)	++ (100%)	+ (100%)	++ (100%)	++ (100%)	++ (100%)	++ (100%)	++ (100%)	++ (100%)	- (100%)	++ (100%)	++ (100%)	- (80%)	++ (100%)	+ (100%)	++ (100%)	++ (100%)	++ (100%)
Gluta	- (100%)	- (100%)	- (100%)	- (100%)	- (100%)	- (100%)	- (100%)	- (100%)	- (100%)	- (100%)	- (100%)	- (100%)	- (100%)	- (100%)	- (100%)	- (100%)	- (100%)	+ (50%)	- (100%)	- (100%)	- (100%)	++ (100%)	- (100%)	++ (100%)	- (100%)	- (100%)	- (100%)
Mesac	- (100%)	- (100%)	- (100%)	- (100%)	- (100%)	- (100%)	- (100%)	- (100%)	- (100%)	- (100%)	- (100%)	- (100%)	- (100%)	- (100%)	- (100%)	- (100%)	- (100%)	+ (50%)	- (100%)	- (100%)	- (100%)	++ (100%)	- (100%)	+ (100%)	- (100%)	- (100%)	- (100%)
D-His	- (100%)	- (100%)	- (100%)	- (100%)	- (100%)	- (100%)	- (100%)	- (100%)	- (100%)	- (100%)	- (100%)	- (100%)	- (100%)	- (100%)	- (100%)	- (100%)	- (100%)	+ (50%)	- (100%)	- (100%)	- (100%)	- (100%)	++ (100%)	- (100%)	- (100%)	- (100%)	- (100%)
NTI	++ (100%)	++ (100%)	++ (100%)	++ (100%)	++ (100%)	++ (80%)	+ (100%)	++ (100%)	++ (71,4%)	++ (100%)	++ (100%)	+ (66,7%)	++ (100%)	+ (100%)	++ (100%)	++ (100%)	- (100%)	++ (50%)	++ (100%)	++ (100%)	++ (93,8%)	+ (70%)	+ (100%)	+ (100%)	- (100%)	- (100%)	- (100%)
NTA	++ (100%)	++ (100%)	++ (100%)	++ (100%)	++ (100%)	++ (80%)	+ (100%)	++ (100%)	++ (71,4%)	++ (100%)	++ (100%)	++ (50%)	++ (100%)	+ (100%)	++ (100%)	++ (100%)	- (100%)	++ (50%)	++ (100%)	++ (100%)	++ (93,8%)	+ (70%)	+ (100%)	++ (50%)	- (100%)	- (100%)	- (100%)
PCA	- (100%)	- (100%)	- (100%)	- (100%)	- (100%)	- (100%)	- (100%)	- (100%)	- (57,1%)	- (71,4%)	- (90,9%)	- (100%)	- (100%)	- (100%)	- (100%)	- (100%)	- (100%)	- (100%)	- (100%)	- (100%)	- (100%)	- (100%)	- (100%)	+ (50%)	- (100%)	- (100%)	- (100%)
VP	+ (100%)	- (100%)	+ (75%)	+ (83,3%)	+ (100%)	+ (100%)	+ (100%)	+ (100%)	- (57,1%)	- (57,1%)	+ (63,6%)	+ (100%)	- (100%)	+ (100%)	- (100%)	- (100%)	- (100%)	+ (100%)	+ (100%)	+ (100%)	+ (93,8%)	++ (60%)	++ (100%)	++ (100%)	- (100%)	- (100%)	+ (100%)
Urease	+ (83,3%)	+ (100%)	+ (100%)	+ (100%)	+ (100%)	+ (100%)	+ (100%)	+ (100%)	+ (100%)	+ (100%)	+ (100%)	+ (100%)	+ (100%)	+ (100%)	+ (100%)	+ (100%)	- (100%)	+ (100%)	+ (100%)	+ (100%)	+ (75%)	+ (100%)	+ (100%)	+ (100%)	- (100%)	- (100%)	+ (100%)
H2S	- (100%)	- (100%)	- (100%)	- (100%)	- (100%)	- (100%)	- (100%)	- (100%)	- (100%)	- (100%)	- (100%)	+ (83,3%)	- (100%)	+ (100%)	+ (100%)	+ (100%)	- (100%)	+ (50%)	- (100%)	- (100%)	+ (75%)	+ (100%)	++ (100%)	++ (50%)	- (100%)	+ (100%)	- (100%)

	B. abortus	*B. melitensis*	*B. suis*	*B. ovis*	*B. canis*	*B. neotomae*	*B. ceti*	*B. pinnipedialis*	*B. microti*	*B. inopinata*	*Ochrobactrum* spp.	*Acinetobacter lwoffi*	*Y. enterocolitica* O:9	*Brucella* unknown
DANA	++ (100%)	++ (100%)	++ (88,9%)	++ (100%)	++ (100%)	- (100%)	++ (100%)	++ (100%)	++ (100%)	++ (100%)	++ (100%)	- (100%)	- (100%)	- (100%)
GNA	++ (100%)	++ (100%)	++ (88,9%)	++ (100%)	++ (100%)	- (100%)	++ (100%)	++ (100%)	++ (100%)	++ (100%)	++ (100%)	- (100%)	- (100%)	- (100%)
LNA	+ (96,7%)	+ (100%)	+ (100%)	+ (100%)	+ (100%)	- (100%)	+ (100%)	+ (93,8%)	+ (100%)	++ (100%)	+ (100%)	++ (100%)	+ (100%)	+ (100%)
KNA	+ (76,7%)	++ (87,9%)	++ (50%)	+ (100%)	++ (100%)	- (100%)	++ (100%)	++ (87,5%)	++ (100%)	++ (100%)	++ (100%)	++ (100%)	++ (100%)	++ (100%)
GPNA	- (100%)	++ (63,6%)	++ (61,1%)	- (100%)	++ (100%)	- (100%)	- (100%)	- (93,8%)	+ (100%)	++ (100%)	+ (50%)	- (100%)	- (100%)	- (100%)
AFPNA	- (100%)	+ (93,9%)	+ (88,9%)	- (100%)	+ (50%)	- (100%)	- (100%)	- (100%)	- (90%)	+ (100%)	- (100%)	- (100%)	- (100%)	- (100%)
ENAOH	- (100%)	- (100%)	- (94,4%)	- (100%)	- (100%)	- (100%)	- (100%)	- (100%)	- (100%)	- (100%)	++ (100%)	- (100%)	- (100%)	- (100%)
PYRNA	- (100%)	- (100%)	- (100%)	- (100%)	- (100%)	- (100%)	- (100%)	- (100%)	- (100%)	- (100%)	++ (100%)	- (100%)	- (100%)	- (100%)
HP	++ (96,7%)	++ (100%)	++ (100%)	++ (100%)	++ (100%)	++ (100%)	++ (100%)	++ (100%)	++ (100%)	++ (100%)	++ (100%)	- (100%)	- (100%)	++ (100%)
lL	- (100%)	- (100%)	- (100%)	- (100%)	- (100%)	- (100%)	- (100%)	- (100%)	- (100%)	- (100%)	+ (50%)	- (100%)	- (100%)	- (100%)
K	++ (93,3%)	++ (100%)	++ (66,7%)	++ (100%)	++ (75%)	- (100%)	++ (100%)	++ (87,5%)	++ (100%)	++ (100%)	++ (100%)	++ (100%)	++ (100%)	++ (100%)
RR	+ (66,7%)	+ (51,5%)	++ (77,8%)	++ (100%)	++ (75%)	+ (100%)	+ (100%)	- (62,5%)	++ (50%)	++ (100%)	++ (100%)	++ (100%)	++ (100%)	+ (100%)
GG	++ (100%)	++ (100%)	++ (100%)	++ (100%)	++ (100%)	- (100%)	++ (100%)	++ (100%)	++ (100%)	++ (100%)	++ (100%)	++ (100%)	++ (100%)	++ (100%)
P	++ (100%)	++ (100%)	++ (100%)	++ (100%)	++ (100%)	++ (100%)	++ (100%)	++ (100%)	++ (100%)	++ (100%)	++ (100%)	- (100%)	- (100%)	++ (100%)
AcGK	- (96,7%)	+ (57,6%)	+ (100%)	++ (100%)	++ (75%)	+ (100%)	- (100%)	- (100%)	- (90%)	++ (100%)	+ (100%)	+ (100%)	- (100%)	+ (50%)
AFPA	+ (96,7%)	++ (100%)	++ (100%)	+ (50%)	++ (100%)	++ (100%)	++ (100%)	++ (56,3%)	+ (80%)	++ (100%)	++ (50%)	++ (100%)	++ (100%)	++ (100%)
N	+ (90%)	+ (90,9%)	++ (61,1%)	+ (75%)	+ (75%)	+ (100%)	+ (100%)	+ (62,5%)	- (100%)	++ (100%)	- (100%)	+ (100%)	- (100%)	++ (100%)
W	++ (86,7%)	++ (90,9%)	++ (77,8%)	- (100%)	- (100%)	++ (100%)	++ (100%)	++ (100%)	++ (90%)	++ (100%)	++ (50%)	+ (100%)	- (100%)	- (100%)
EH	+ (90%)	+ (93,9%)	++ (55,6%)	- (100%)	+ (75%)	+ (100%)	++ (100%)	- (81,3%)	+ (90%)	++ (100%)	+ (100%)	- (100%)	- (100%)	+ (100%)
AcKA	- (100%)	+ (60,6%)	+ (100%)	++ (75%)	++ (50%)	+ (100%)	- (100%)	- (100%)	- (100%)	++ (100%)	+ (100%)	- (100%)	- (100%)	- (100%)
dAdA	+ (100%)	- (57,6%)	+ (83,3%)	- (100%)	+ (50%)	- (100%)	+ (100%)	+ (81,3%)	+ (100%)	++ (100%)	++ (100%)	- (100%)	- (100%)	- (100%)
V	- (100%)	- (100%)	- (61,1%)	- (100%)	- (100%)	- (100%)	- (100%)	- (93,8%)	- (100%)	- (100%)	- (100%)	+ (100%)	- (100%)	- (100%)
I	- (100%)	- (100%)	- (100%)	- (100%)	- (100%)	- (100%)	- (100%)	- (100%)	- (100%)	- (100%)	- (100%)	+ (100%)	- (100%)	- (100%)
VTS	- (96,7%)	+ (66,7%)	+ (55,6%)	+ (100%)	+ (50%)	- (100%)	- (100%)	- (56,3%)	+ (50%)	++ (100%)	+ (100%)	+ (100%)	- (100%)	+ (100%)
D	- (100%)	- (100%)	- (100%)	- (100%)	- (100%)	- (100%)	- (100%)	- (100%)	- (100%)	- (100%)	- (100%)	++ (100%)	- (100%)	- (100%)
E	- (100%)	- (100%)	- (100%)	- (100%)	- (100%)	- (100%)	- (100%)	- (100%)	- (100%)	- (100%)	- (100%)	++ (100%)	+ (100%)	- (100%)
Pyr	- (100%)	- (100%)	- (100%)	- (100%)	- (100%)	- (100%)	- (100%)	- (100%)	- (100%)	- (100%)	++ (100%)	- (100%)	++ (100%)	- (100%)
βA	- (100%)	- (100%)	- (100%)	- (100%)	- (100%)	- (100%)	- (100%)	- (100%)	- (100%)	- (100%)	+ (100%)	- (100%)	- (100%)	- (100%)
V4M	- (100%)	- (100%)	- (100%)	- (100%)	- (100%)	- (100%)	- (100%)	- (100%)	- (100%)	- (100%)	- (100%)	- (100%)	- (100%)	- (100%)
PepK	- (100%)	- (100%)	- (100%)	- (100%)	- (100%)	- (100%)	- (100%)	- (100%)	- (100%)	- (100%)	- (100%)	- (100%)	- (100%)	- (100%)
BISPH7	- (80%)	- (66,7%)	++ (66,7%)	+ (50%)	+ (100%)	- (100%)	+ (100%)	++ (56,3%)	++ (100%)	++ (100%)	++ (100%)	- (100%)	++ (100%)	+ (100%)
PHOS7	- (83,3%)	+ (100%)	++ (50%)	- (75%)	+ (75%)	- (100%)	+ (100%)	+ (100%)	++ (100%)	++ (100%)	+ (100%)	+ (100%)	++ (100%)	+ (100%)
aGLU7	- (100%)	+ (60,6%)	+ (61,1%)	- (100%)	+ (100%)	+ (100%)	- (100%)	+ (50%)	++ (100%)	++ (100%)	++ (100%)	- (100%)	- (100%)	- (100%)
aMAL7	- (100%)	- (100%)	- (83,3%)	- (100%)	- (100%)	- (100%)	- (100%)	- (100%)	+ (100%)	+ (100%)	+ (100%)	- (100%)	- (100%)	- (100%)
aGLU5	- (100%)	- (100%)	+ (83,3%)	- (100%)	+ (100%)	- (100%)	- (100%)	- (56,3%)	++ (100%)	++ (100%)	+ (100%)	- (100%)	++ (100%)	- (100%)
aXYL7	- (100%)	- (100%)	+ (94,4%)	- (100%)	+ (50%)	- (100%)	- (100%)	- (87,5%)	+ (100%)	++ (100%)	+ (100%)	- (100%)	- (100%)	- (100%)
CHIT7	- (96,7%)	+ (97%)	+ (88,9%)	- (100%)	++ (50%)	+ (100%)	- (100%)	+ (100%)	++ (100%)	++ (100%)	++ (100%)	- (100%)	++ (100%)	++ (100%)
EROL	++ (100%)	++ (100%)	++ (77,8%)	- (100%)	+ (100%)	++ (100%)	++ (100%)	++ (50%)	++ (100%)	++ (100%)	++ (100%)	- (100%)	- (100%)	++ (100%)
L-FUC	- (53,3%)	++ (100%)	+ (100%)	- (100%)	++ (50%)	- (100%)	++ (100%)	- (75%)	++ (100%)	++ (100%)	++ (100%)	- (100%)	- (100%)	+ (100%)
GLUCY	++ (100%)	++ (100%)	++ (100%)	+ (100%)	++ (100%)	++ (100%)	++ (100%)	++ (100%)	++ (100%)	++ (100%)	++ (100%)	- (100%)	++ (100%)	++ (100%)
D-RIB	++ (100%)	- (87,9%)	++ (100%)	- (100%)	++ (50%)	- (100%)	++ (100%)	- (100%)	++ (100%)	++ (100%)	++ (100%)	- (100%)	++ (100%)	+ (100%)
D-ARA	- (80%)	+ (60,6%)	- (77,8%)	- (100%)	+ (100%)	- (100%)	- (100%)	- (100%)	+ (60%)	++ (100%)	++ (100%)	- (100%)	- (100%)	- (100%)
D-GLU	++ (100%)	++ (93,9%)	++ (100%)	- (100%)	++ (100%)	++ (100%)	++ (100%)	- (62,5%)	++ (100%)	++ (100%)	++ (100%)	- (100%)	++ (100%)	++ (100%)
L-ARA	++ (80%)	++ (93,9%)	++ (55,6%)	+ (50%)	+ (100%)	++ (100%)	++ (100%)	- (87,5%)	++ (100%)	++ (100%)	++ (100%)	- (100%)	++ (100%)	- (100%)
DOGAL	- (100%)	- (87,9%)	- (100%)	- (100%)	- (100%)	- (100%)	- (100%)	- (100%)	- (100%)	- (100%)	- (100%)	- (100%)	- (100%)	- (100%)
D-GAL	++ (100%)	++ (93,9%)	++ (55,6%)	- (100%)	+ (50%)	++ (100%)	++ (100%)	- (87,5%)	++ (100%)	++ (100%)	++ (100%)	- (100%)	++ (100%)	+ (100%)
D-XYL	+ (66,7%)	++ (100%)	+ (55,6%)	- (100%)	++ (50%)	++ (100%)	++ (100%)	- (100%)	++ (100%)	++ (100%)	++ (100%)	- (100%)	++ (100%)	++ (100%)
D-TAL	+ (66,7%)	+ (60,6%)	+ (66,7%)	- (100%)	+ (50%)	++ (100%)	++ (100%)	- (87,5%)	++ (100%)	- (100%)	++ (100%)	- (100%)	- (100%)	- (100%)
D-TOL	++ (80%)	- (100%)	+ (66,7%)	++ (50%)	++ (100%)	- (100%)	++ (100%)	+ (75%)	++ (100%)	++ (100%)	++ (100%)	- (100%)	- (100%)	- (100%)
ADON	++ (93,3%)	++ (81,8%)	++ (55,6%)	+ (100%)	- (100%)	++ (100%)	++ (100%)	++ (75%)	++ (100%)	- (100%)	++ (100%)	- (100%)	- (100%)	- (100%)
SUCR	- (100%)	- (100%)	++ (77,8%)	- (100%)	++ (100%)	- (100%)	- (100%)	+ (87,5%)	++ (100%)	++ (100%)	++ (100%)	- (100%)	++ (100%)	- (100%)
MALTO	- (100%)	- (100%)	++ (77,8%)	- (100%)	++ (100%)	- (100%)	- (100%)	++ (62,5%)	++ (100%)	++ (100%)	++ (100%)	- (100%)	- (100%)	- (100%)
D-FRU	+ (53,3%)	++ (100%)	++ (100%)	- (100%)	++ (100%)	++ (100%)	++ (100%)	+ (62,5%)	++ (100%)	++ (100%)	++ (100%)	- (100%)	++ (100%)	++ (100%)
DNSOL	- (100%)	- (100%)	- (100%)	- (100%)	- (100%)	- (100%)	- (100%)	- (100%)	- (100%)	- (100%)	++ (100%)	- (100%)	- (100%)	- (100%)
LYXA	- (100%)	- (100%)	+ (88,9%)	- (100%)	++ (100%)	++ (100%)	++ (100%)	- (100%)	++ (100%)	++ (100%)	++ (100%)	- (100%)	- (100%)	++ (100%)
PAL	- (100%)	- (100%)	++ (77,8%)	- (100%)	++ (100%)	- (100%)	- (100%)	+ (50%)	++ (100%)	++ (100%)	++ (100%)	- (100%)	- (100%)	- (100%)
INOL	- (60%)	+ (90,9%)	- (100%)	- (100%)	- (100%)	- (100%)	- (100%)	- (100%)	- (100%)	++ (100%)	++ (100%)	- (100%)	++ (100%)	- (100%)
INON	- (73,3%)	- (100%)	+ (55,6%)	- (100%)	++ (50%)	- (100%)	++ (100%)	+ (87,5%)	++ (100%)	- (100%)	++ (100%)	- (100%)	++ (100%)	++ (100%)
Galli	++ (100%)	++ (100%)	++ (100%)	++ (100%)	++ (100%)	++ (100%)	++ (100%)	++ (100%)	++ (100%)	++ (100%)	++ (100%)	++ (100%)	++ (100%)	++ (100%)
diLac	++ (100%)	++ (100%)	++ (100%)	++ (50%)	++ (100%)	++ (100%)	++ (100%)	++ (100%)	++ (100%)	++ (100%)	++ (100%)	++ (100%)	- (100%)	++ (100%)
Acet	- (80%)	- (87,9%)	++ (88,9%)	++ (100%)	++ (100%)	++ (100%)	- (100%)	- (87,5%)	++ (100%)	++ (100%)	++ (100%)	++ (100%)	- (100%)	- (100%)
L-Asn	++ (80%)	++ (100%)	++ (55,6%)	- (100%)	++ (100%)	- (100%)	- (100%)	- (87,5%)	++ (100%)	++ (100%)	++ (100%)	- (100%)	++ (100%)	- (100%)
L-Glu	++ (86,7%)	++ (100%)	++ (66,7%)	++ (50%)	++ (100%)	++ (100%)	++ (100%)	+ (87,5%)	++ (100%)	++ (100%)	++ (100%)	- (100%)	++ (100%)	++ (100%)
AlaGln	++ (86,7%)	++ (69,7%)	+ (100%)	- (100%)	+ (100%)	- (100%)	- (100%)	- (100%)	++ (100%)	++ (100%)	++ (100%)	- (100%)	++ (100%)	++ (100%)
GuaSu	- (73,3%)	- (100%)	- (55,6%)	- (100%)	++ (100%)	- (100%)	++ (100%)	++ (100%)	++ (100%)	- (100%)	- (100%)	- (100%)	- (100%)	- (100%)
L-Cyss	++ (100%)	++ (100%)	++ (100%)	++ (100%)	+ (50%)	++ (100%)	++ (100%)	++ (100%)	++ (100%)	++ (100%)	+ (100%)	- (100%)	- (100%)	++ (100%)
D-Ala	+ (60%)	++ (87,9%)	+ (55,6%)	- (100%)	- (100%)	++ (100%)	- (100%)	- (100%)	++ (60%)	++ (100%)	++ (100%)	++ (100%)	- (100%)	- (100%)
Propn	- (86,7%)	++ (100%)	+ (88,9%)	- (100%)	+ (100%)	++ (100%)	- (100%)	+ (75%)	- (100%)	++ (100%)	++ (100%)	++ (100%)	- (100%)	- (100%)
L-Ala	+ (80%)	++ (100%)	+ (66,7%)	- (100%)	++ (50%)	- (100%)	- (100%)	- (100%)	++ (100%)	++ (100%)	++ (100%)	++ (100%)	++ (100%)	- (100%)
βHBut	- (100%)	- (100%)	+ (100%)	- (100%)	++ (100%)	++ (100%)	- (100%)	- (100%)	++ (60%)	++ (100%)	++ (100%)	++ (100%)	- (100%)	- (100%)
D-Asn	- (73,3%)	- (51,5%)	- (100%)	- (100%)	- (100%)	- (100%)	- (100%)	- (100%)	- (100%)	- (100%)	+ (100%)	- (100%)	- (100%)	- (100%)
L-Arg	- (93,3%)	- (100%)	++ (66,7%)	- (100%)	++ (100%)	- (100%)	- (100%)	- (100%)	++ (100%)	++ (100%)	++ (100%)	- (100%)	- (100%)	+ (100%)
AcArg	- (100%)	- (100%)	++ (66,7%)	- (100%)	++ (100%)	- (100%)	- (100%)	- (100%)	+ (80%)	++ (100%)	++ (100%)	- (100%)	- (100%)	+ (100%)
Glyx	- (100%)	- (93,9%)	- (66,7%)	- (100%)	+ (50%)	- (100%)	- (100%)	- (100%)	- (100%)	++ (100%)	+ (100%)	- (100%)	- (100%)	- (100%)
L-Ser	- (93,3%)	++ (87,9%)	+ (88,9%)	+ (100%)	+ (50%)	- (100%)	- (100%)	- (100%)	++ (100%)	++ (100%)	++ (100%)	- (100%)	++ (100%)	- (100%)
HipArg	- (100%)	- (100%)	++ (55,6%)	- (100%)	+ (100%)	- (100%)	- (100%)	- (100%)	+ (80%)	++ (100%)	++ (100%)	- (100%)	- (100%)	- (100%)
L-Caro	- (60%)	- (100%)	+ (77,8%)	- (100%)	- (100%)	- (100%)	- (100%)	- (100%)	++ (100%)	++ (100%)	++ (100%)	- (100%)	- (100%)	- (100%)
Gly	- (100%)	- (100%)	- (88,9%)	- (100%)	- (100%)	- (100%)	- (100%)	- (100%)	++ (60%)	++ (100%)	+ (100%)	- (100%)	- (100%)	- (100%)
Adipa	- (100%)	- (100%)	+ (88,9%)	- (100%)	+ (50%)	- (100%)	++ (100%)	- (100%)	++ (100%)	- (100%)	+ (100%)	++ (100%)	- (100%)	- (100%)
L-Pro	- (80%)	++ (87,9%)	- (88,9%)	- (100%)	- (100%)	- (100%)	- (100%)	- (75%)	++ (100%)	++ (100%)	++ (100%)	++ (100%)	++ (100%)	- (100%)
D-Pro	- (93,3%)	++ (75,8%)	- (66,7%)	- (100%)	+ (50%)	- (100%)	- (100%)	- (100%)	++ (100%)	++ (100%)	++ (100%)	- (100%)	- (100%)	- (100%)
Fuma	- (66,7%)	- (93,9%)	- (88,9%)	- (100%)	- (100%)	- (100%)	- (100%)	- (100%)	++ (100%)	++ (100%)	+ (100%)	++ (100%)	++ (100%)	- (100%)
D-Ser	- (100%)	++ (81,8%)	- (100%)	- (100%)	- (100%)	- (100%)	- (100%)	- (100%)	- (100%)	- (100%)	- (100%)	- (100%)	- (100%)	- (100%)
Glyc	- (100%)	- (93,9%)	- (55,6%)	- (100%)	++ (50%)	- (100%)	- (100%)	- (100%)	- (100%)	++ (100%)	++ (100%)	- (100%)	- (100%)	- (100%)
Adeni	++ (100%)	++ (100%)	++ (66,7%)	++ (100%)	++ (100%)	- (100%)	++ (100%)	++ (100%)	- (80%)	++ (100%)	+ (100%)	++ (100%)	++ (100%)	++ (100%)
Gluta	- (100%)	- (100%)	- (100%)	- (100%)	+ (50%)	- (100%)	- (100%)	- (100%)	++ (100%)	- (100%)	++ (100%)	- (100%)	- (100%)	- (100%)
Mesac	- (100%)	- (100%)	- (100%)	- (100%)	+ (50%)	- (100%)	- (100%)	- (100%)	++ (100%)	- (100%)	+ (100%)	- (100%)	- (100%)	- (100%)
D-His	- (100%)	- (100%)	- (100%)	- (100%)	+ (50%)	- (100%)	- (100%)	- (100%)	- (100%)	++ (100%)	- (100%)	- (100%)	- (100%)	- (100%)
NTI	++ (86,7%)	++ (93,9%)	++ (72,2%)	- (100%)	++ (50%)	++ (100%)	++ (100%)	++ (93,8%)	+ (70%)	+ (100%)	+ (100%)	- (100%)	- (100%)	- (100%)
NTA	++ (86,7%)	++ (93,9%)	++ (77,8%)	- (100%)	++ (50%)	++ (100%)	++ (100%)	++ (93,8%)	+ (70%)	+ (100%)	++ (50%)	- (100%)	- (100%)	- (100%)
PCA	- (100%)	- (75,8%)	- (100%)	- (100%)	- (100%)	- (100%)	- (100%)	- (100%)	- (100%)	- (100%)	+ (50%)	- (100%)	- (100%)	- (100%)
VP	+ (86,7%)	+ (51,5%)	- (61,1%)	- (100%)	+ (100%)	+ (100%)	+ (100%)	+ (93,8%)	++ (60%)	++ (100%)	++ (100%)	- (100%)	- (100%)	+ (100%)
Urease	+ (96,7%)	+ (97%)	+ (100%)	- (100%)	+ (100%)	+ (100%)	+ (100%)	+ (75%)	+ (100%)	+ (100%)	+ (100%)	- (100%)	- (100%)	+ (100%)
H2S	- (100%)	- (100%)	+ (50%)	- (100%)	+ (50%)	- (100%)	- (100%)	+ (75%)	+ (100%)	++ (100%)	++ (50%)	- (100%)	+ (100%)	- (100%)
