# Supplementary material for: Differential phenotyping of Brucella species using a newly developed semi-automated metabolic system
Source: BMC Microbiol. 2010 Oct 23;10:269. doi: 10.1186/1471-2180-10-269 (PMC2984481; doi:10.1186/1471-2180-10-269)
Supplement: Additional file 8 — Separation of Brucella spp. from clinically relevant bacteria. Relative frequency (%) of positive metabolic activity among Brucella and other bacteria observed for HP, Pyr-βNA (Pyr), urease, and NTA. Four enzymatic reactions which were revealed by the Micronaut-IDS database screening clearly discriminated Brucella from clinically relevant bacteria of other genera. [file 1471-2180-10-269-S8.PDF]

	HP	Pyr	Urease	NTA
B. abortus **bv 1**	**100.0**	0.0	**83.3**	**100.0**
B. abortus **bv 2**	**100.0**	0.0	**100.0**	**100.0**
B. abortus **bv 3**	**100.0**	0.0	**100.0**	**100.0**
B. abortus **bv 4**	**83.3**	0.0	**100.0**	**100.0**
B. abortus **bv 5**	**100.0**	0.0	**100.0**	**100.0**
B. abortus **bv 6**	**100.0**	0.0	**100.0**	**80.0**
B. abortus **bv 7**	**100.0**	0.0	**100.0**	**100.0**
B. abortus **bv 9**	**100.0**	0.0	**100.0**	**100.0**
B. melitensis **bv 1**	**100.0**	0.0	**100.0**	**71.4**
B. melitensis **bv 2**	**100.0**	0.0	**100.0**	**100.0**
B. melitensis **bv 3**	**100.0**	0.0	**100.0**	**100.0**
B. suis **bv 1**	**100.0**	0.0	**100.0**	*50.0*
B. suis **bv 2**	**100.0**	0.0	**100.0**	**100.0**
B. suis **bv 3**	**100.0**	0.0	**100.0**	**100.0**
B. suis **bv 4**	**100.0**	0.0	**100.0**	**100.0**
B. suis **bv 5**	**100.0**	0.0	**100.0**	**100.0**
B. ovis	**100.0**	0.0	0.0	0.0
B. canis	**100.0**	0.0	**100.0**	*50.0*
B. neotomae	**100.0**	0.0	**100.0**	**100.0**
B. ceti	**100.0**	0.0	**100.0**	**100.0**
B. pinnipedialis	**100.0**	0.0	**75.0**	**93.8**
B. microti	**100.0**	0.0	**100.0**	**70.0**
B. inopinata	**100.0**	0.0	**100.0**	**100.0**
Enterococcus gallinarum	1.0	**99.9**	0.1	0.0
Enterococcus faecalis	1.0	**99.0**	0.1	0.0
Enterococcus faecium	12.0	**99.0**	0.1	0.0
Citrobacter koseri	1.0	**99.0**	10.0	**100.0**
Staphylococcus haemolyticus	0.1	**99.9**	1.0	**89.0**
Enterobacter aerogenes	1.0	**99.0**	4.0	**100.0**
Citrobacter freundii	1.0	**98.0**	*34.0*	**100.0**
Klebsiella oxytoca	0.1	**92.0**	**93.0**	**100.0**
Klebsiella pneumoniae **sp.** *pneumoniae*	0.1	**96.0**	**96.0**	**100.0**
Stenotrophomonas maltophilia	6.0	4.0	1.0	12.0
Acinetobacter species **II**	0.1	0.1	0.1	0.0
Streptococcus agalactiae	0.1	0.1	0.1	0.0
Streptococcus pneumoniae	15.0	20.0	0.1	0.0
Providencia stuartii	0.1	0.1	30.0	**100.0**
Enterobacter cloacae	1.0	11.0	9.0	**100.0**
Escherichia coli	1.0	1.0	0.1	**100.0**
Salmonella species	0.1	0.1	0.1	**100.0**
Pseudomonas aeruginosa	1.0	8.0	*54.0*	**97.0**
Morganella morganii	1.0	1.0	**98.0**	**100.0**
Proteus mirabilis	1.0	1.0	**99.0**	**100.0**
Proteus vulgaris	1.0	1.0	**99.9**	**100.0**
Staphylococcus epidermidis	0.1	1.0	**98.0**	**92.0**
Staphylococcus aureus	0.1	*32.0*	**92.0**	**96.0**
Serratia marcescens	**90.0**	**92.0**	12.0	**100.0**
Plesiomonas shigelloides	**80.0**	0.1	10.0	**100.0**
Streptococcus pyogenes	1.0	**99.0**	0.1	0.0
